# A multi-animal tracker for studying complex behaviors

**DOI:** 10.1186/s12915-017-0363-9

**Published:** 2017-04-06

**Authors:** Eyal Itskovits, Amir Levine, Ehud Cohen, Alon Zaslaver

**Affiliations:** 1 Department of Genetics, The Silberman Institute of Life Science, Edmond J. Safra Campus, The Hebrew University of Jerusalem, Jerusalem, 91904 Israel; 2 School of Computer Science and Engineering, Hebrew University, Jerusalem, Israel; 3 Biochemistry and Molecular Biology, The Institute for Medical Research Israel – Canada (IMRIC), School of Medicine, The Hebrew University of Jerusalem, Jerusalem, 91120 Israel

**Keywords:** Multi-animal tracking, Image analyses, Chemotaxis, *C. elegans*, Locomotion

## Abstract

**Background:**

Animals exhibit astonishingly complex behaviors. Studying the subtle features of these behaviors requires quantitative, high-throughput, and accurate systems that can cope with the often rich perplexing data.

**Results:**

Here, we present a Multi-Animal Tracker (MAT) that provides a user-friendly, end-to-end solution for imaging, tracking, and analyzing complex behaviors of multiple animals simultaneously. At the core of the tracker is a machine learning algorithm that provides immense flexibility to image various animals (e.g., worms, flies, zebrafish, etc.) under different experimental setups and conditions. Focusing on *C. elegans* worms, we demonstrate the vast advantages of using this MAT in studying complex behaviors. Beginning with chemotaxis, we show that approximately 100 animals can be tracked simultaneously, providing rich behavioral data. Interestingly, we reveal that worms’ directional changes are biased, rather than random – a strategy that significantly enhances chemotaxis performance. Next, we show that worms can integrate environmental information and that directional changes mediate the enhanced chemotaxis towards richer environments. Finally, offering high-throughput and accurate tracking, we show that the system is highly suitable for longitudinal studies of aging- and proteotoxicity-associated locomotion deficits, enabling large-scale drug and genetic screens.

**Conclusions:**

Together, our tracker provides a powerful and simple system to study complex behaviors in a quantitative, high-throughput, and accurate manner.

**Electronic supplementary material:**

The online version of this article (doi:10.1186/s12915-017-0363-9) contains supplementary material, which is available to authorized users.

## Background

Animal behavior is rich and complex [1]. It spans a tremendously wide range of phenotypes such as sleep, mating, food search, and fighting. In its early days, this fascinating field was dominated by classical field studies involving visual inspection and hand-written documentation. The discipline has considerably evolved and nowadays is known as “computational ethology” [2]. Technology advances are now replacing the laborious manual work and scientists employ sophisticated computational approaches to generate accurate quantitative understanding of the detail-rich complex behaviors.

In the last few decades, significant advances in genetic- and neural-related techniques have been achieved, and animal model systems have become popular to study complex behaviors under lab-controlled environments. These animal models range from simple invertebrates, such as worms and flies, to mammalian models, including mice and monkeys [2].

Of particular interest is the small roundworm *C. elegans* [3]. Among the many advantages that this nematode offers are its short generation time, the easy and inexpensive handling, and the availability of its fully-reconstructed neural system consisting of only 302 neurons [4]. *C. elegans* animals also show remarkably complex behaviors, including mating [5], roaming and foraging [6], lethargus [7], and taxis towards various stimuli such as preferred temperature [8], magnetic fields [9], and food [10–12]. Over the years, large genetic screens have identified a vast set of mutations in genes that modulate these behavioral outputs, enabling a mechanistic insight into these complex behaviors [13, 14].

In addition, a growing interest is directed towards quantitative measures of aging and proteotoxicity-associated physiological decline. *C. elegans* is an appealing model organism to study these processes as many human neurodegenerative diseases that result from aggregation of proteotoxic proteins can be recapitulated in the worms’ muscular and nervous systems [15]. These diseases are often associated with deteriorating locomotion abilities, culminating eventually in complete paralysis. It therefore becomes an important, yet extremely challenging, task to quantify minute locomotive changes in a high-throughput manner.

To study such complex behaviors, numerous tracking systems that image freely-moving animals have been developed. These systems extract various fine locomotion features, such as animal posture, undulation properties, speed, and attraction or repulsion towards or away from stimulants [16–23]. To support high-throughput studies, some of these systems were designed to track multiple worms at a time. This sort of tracking is particularly challenging as accurate extraction of genuine worm entities from ‘noisy’ background is difficult. Moreover, frequent animal collisions preclude extraction of long individual tracks.

Here, we present a novel Multi-Animal Tracker (MAT), an end-to-end, user-friendly solution for imaging, tracking, and analyzing complex behaviors. At the heart of the tracker is a machine learning algorithm; thus, the tracker makes no prior assumptions regarding the size or the shape of the tracked animals and hence is compatible in studying virtually any animal of interest. We also included built-in functions that allow non-programmers to analyze and visualize the immense data. We demonstrate its high-compatibility tracking various animals, and its powerful abilities studying complex behaviors in *C. elegans* animals in an accurate and high-throughput fashion.

## Results

In the following sections, we demonstrate the vast uses of the novel MAT to studying complex behaviors. We begin by describing the packages included in the software suite and then provide experimental evidence, including novel gained insights, for the extensive usage of this MAT in studying chemotaxis and sensory integration, as well as aging- and proteotoxicity-associated locomotion decline.

### The new MAT provides an end-to-end solution for tracking animals – from video acquisition and track identification to advanced analyses

**Fig. 1:**
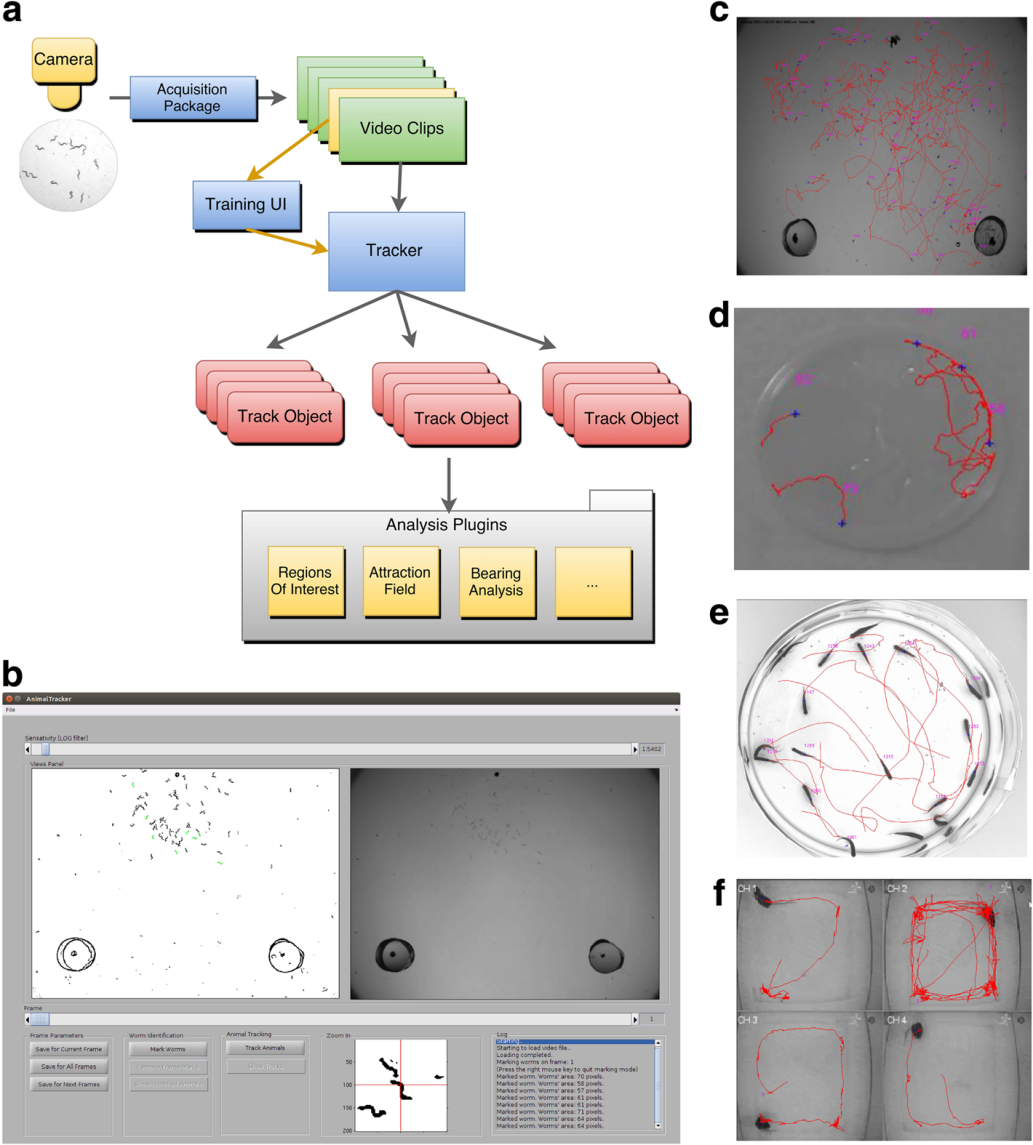
A novel end-to-end Multi-Animal Tracker software allows recording, tracking, and analyzing multiple animals at a time. **a** Flowchart describing the sequential usage of the different packages included in the software suite. **b** A simple, user-friendly Graphical User Interface is provided to analyze tracks of multiple animals throughout a movie recording typically consisting of thousands of frames. The tracking software uses a machine learning algorithm to identify animal instances. The user is asked to ‘teach’ the software what is an animal by clicking on several animal instances. **c**–**f** The software is able to track and analyze the movement of different animals. Shown are representative tracks extracted from movies of behaving (**c**) worms, (**d**) flies, (**e**) zebrafish, and (**f**) mice

Our novel MAT includes three software modules, namely (1) video acquisition, (2) track extraction, and (3) advanced functions for analyzing complex behaviors (Fig. 1a). All packages are based on MATLAB (MathWorks^©^ Inc.) and a detailed guideline for installing and using the different modules is provided in the accompanying Additional file 1.

The ‘*AnimalsRecorder*’ module allows capturing of time-lapse movies in a variety of video formats. This package circumvents the need to rely on commercial software that often interfaces with exclusive cameras and setups. The user can easily modulate various imaging parameters such as recording duration, number of frames, and exposure time.

The main module in the software is ‘*AnimalsTracker*’. This module uses a machine learning algorithm to identify individual animals and extracts their continuous tracks over long temporal recordings, typically consisting thousands of frames. The Graphical User Interface (GUI) in this module displays several panels that allow scrolling between the different frames, and visually comparing the original image to a filtered one (Fig. 1b). This feature provides a simple and convenient mean to inspect and evaluate how well the tracker extracts the observed entities from their background in each frame. The user can quickly adjust the extraction sensitivity between different frames. Once satisfied with the entities extraction, the user is asked to mark individual animals in order to train the machine learning algorithm to correctly identify them.

The additional module, ‘*AnalysesPlugins*’, contains several useful built-in analyses functions; some of these functions are basic analysis tools, but others offer advanced toolkits that allow non-specialist programmers to sophisticatedly analyze the complex behavioral paradigms. These tools are further described below.

### A machine learning algorithm extracts accurate tracks of multiple animals over long time periods

**Fig. 2:**
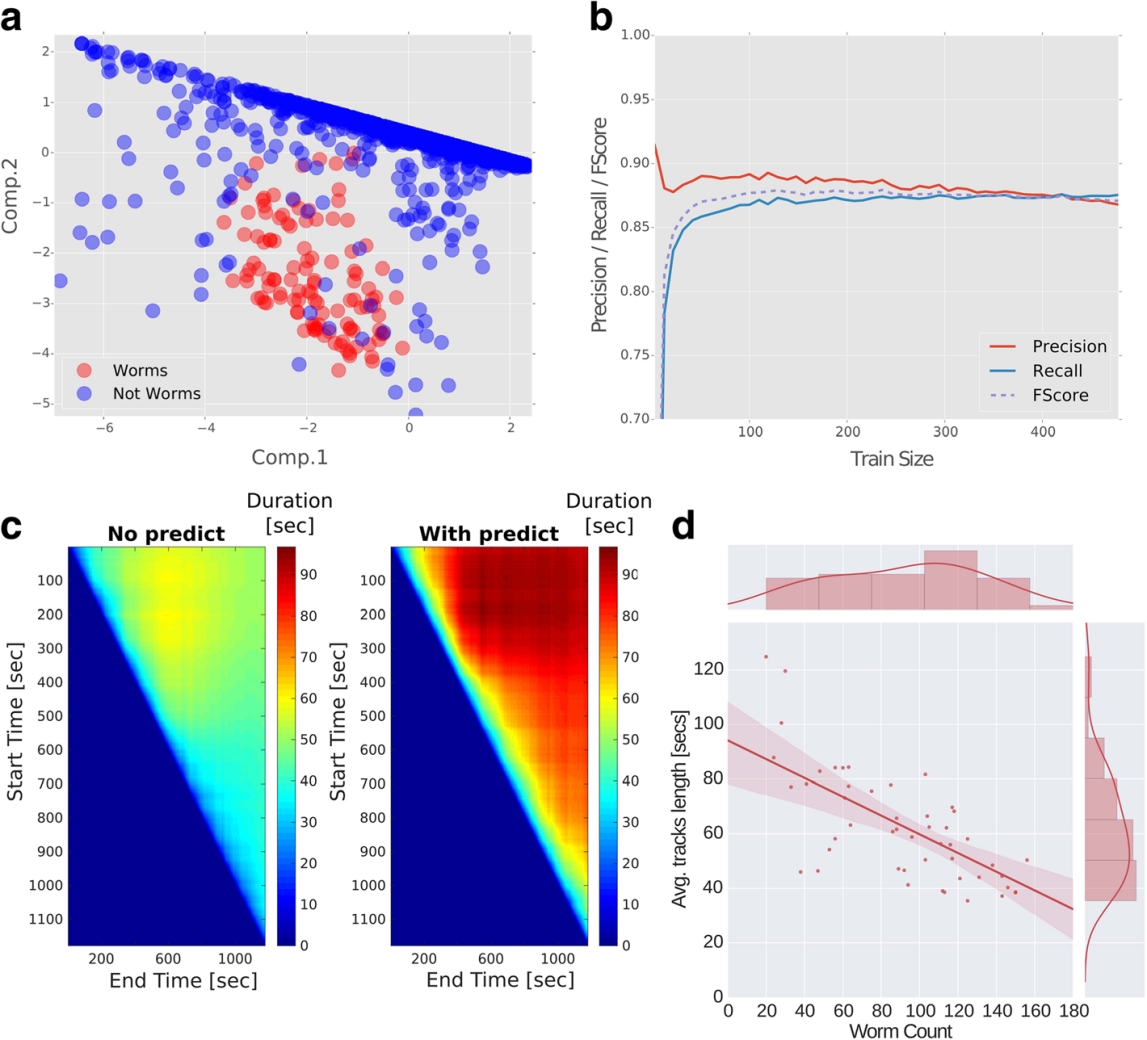
The tracking software successfully extracts trajectories of multiple animals at a time despite background noise. **a** Principal component analysis over all detected animal instances in a single frame shows that worm entities are faithfully segregated from background entities. For example, during long temporal recordings, drop condensation on the lid and trail marks contribute to the identification of approximately 2000 erroneous entities per frame. The number of genuine animal entities, however, remains constant (around 100) throughout the movie (see also Additional file 6: Figure S1). Thus, the software successfully learns the crucial features to detect animal entities and discards background noise. **b** Accuracy of worm detection as a function of the training size. The precision (fraction of actual worms out of total entities classified as worms) reaches nearly 90%, and recall (out of all worms genuinely found on the plate, how many are indeed classified as worms) reaches approximately 85%. Fscore is the harmonic average of the two parameters (precision and recall); as few as 50 worms are sufficient to reach these accuracies (see Methods for details how these parameters were obtained). **c** Implementing a variant of a Kalman predictor significantly enhances track length. Shown are the lengths (in seconds) of all tracks without implementing the predictor (left), and when using the predictor (right). The y-axis and x-axis denote the start and end time, respectively, of the period the tracks lengths were averaged on. Shown are all tracks identified throughout the movie. Significant longer tracks are extracted if implementing the predictor (80 ± 0.54 [s] compared to without the predictor 58 ± 0.3 [s], in the period of the experiment that yielded the longest tracks, *P* < 0.001). **d** There is a trade-off between the number of worms loaded on the assay plate and the average length of detected tracks. The more worms loaded the shorter are the continuously detected tracks. Up to 200 worms can be assayed simultaneously in a field of view of 4 × 4 cm if not requiring extraction of long continuous tracks



At the core of the tracker is a machine learning algorithm which provides an immense flexibility to image and track virtually any moving animal, including worms, flies, zebrafish, and mice (Fig. 1c–f, and Additional file 2: Movie S1, Additional file 3: Movie S2, Additional file 4: Movie S3, Additional file 5: Movie S4). The user is not required to explicitly provide animal features. Rather, the user ‘trains’ the tracker by clicking on a small number of animal entities from the assay images using a user-friendly GUI (Fig. 1b). The tracker learns the animals’ features based on the user’s picks, and uses this information to build a discriminative model between animal entities and background ‘noise’ (Fig. 2a). Moreover, the machine learning algorithm is insensitive to different acquisition parameters such as resolution, contrast, frame rate, color depth, etc. The tracker successfully distinguishes (with a configurable parameter for an approximate false negative rate, β, set to 0.01 by default, see Methods) between animals and background ‘noise’ that typically accumulates during long experiments (Additional file 6: Figure S1a). As few as a dozen clicks on animal entities from the first, mid, and last frames of the movie are sufficient for accurate extraction of animal trajectories throughout thousands of frames. In fact, accuracy analyses show that the tracker achieves a precision of approximately 0.9 and recall of over 0.85 (Fig. 2b, see Methods for details).

**Additional file 2: Movie S1.** The Multi-Animal Tracker can track > 100 worms at a time. (AVI 62803 kb)




**Additional file 3: Movie S2.** The Multi-Animal Tracker can track multiple flies at a time. (MP4 11655 kb)




**Additional file 4: Movie S3.** The Multi-Animal Tracker can track multiple zebrafish at a time. (MP4 39896 kb)




**Additional file 5: Movie S4.** The Multi-Animal Tracker can track multiple mice at a time. The machine learning algorithm, together with the Kalman predictor, filter out possible noise, such as user intervention, during the experiment. (MP4 18744kb)


A major obstacle in maintaining long tracks of individual animals is the frequent collisions between them. To overcome this, we implemented a simple variation of the Kalman filter [24], which predicts the future position of the colliding animals based on previously observed motility features (e.g., velocity, acceleration; see Methods for implementation). Implementing such a predictor significantly improves detection following animal collision; for example, implementing the predictor reduces the cases where none of the animal trajectories could be resolved following collision to approximately 2% only (whereas as many as 46% of the collision events could not be resolved if not implementing the predictor; Additional file 6: Figure S1b). Moreover, the prediction also significantly extends the tracks’ length as it compensates in cases where animal segmentation fails to identify a genuine animal (Fig. 2c, Additional file 7: Figure S2). Overall, this allows the tracker to maintain identification of individual animals over long imaging time periods (Additional file 6: Figure S1c, Additional file 7: Figure S2, Additional file 8: Movie S5, Additional file 9: Movie S6, Additional file 10: Movie S7).



**Additional file 8: Movie S5.** An exemplary movie extracting tracks of swimming zebrafish that does not implement neither the machine learning (ML) algorithms nor the Kalman predictor. Without using these features, trajectory extraction is poor despite the fact that animals are easily detected due to a high contrast with the background. (MP4 39899 kb)




**Additional file 9: Movie S6.** An exemplary movie extracting tracks of swimming zebrafish analyzed by implementing ML algorithms only (the Kalman predictor is not used in these analyses). While segmentation and trajectory extraction is improved when compared to analyses that use neither of them, the results still fall short if compared to analyses that use both (the ML and Kalman predictor). (MP4 33474 kb)




**Additional file 10: Movie S7.** An exemplary movie extracting tracks of swimming zebrafish analyzed by implementing both ML algorithms and the Kalman predictor. Using both features significantly improves segmentation and trajectory extraction. (MP4 35674 kb)


Clearly, collision events become more probable as animal density increases, thereby limiting the time of efficient tracking. In our experimental setups (circular, 50 mm diameter field of view), we found a negative linear correlation (ϱ = −0.7, *P* < 0.001) between the number of worms on the experimental plate and the average duration of tracking (Fig. 2d). In addition, the implemented Kalman-type predictor aids ignoring potential interference during the experiment. For example, the software discounts transient user interventions, such as manual handling of mice during the course of the experiment, and proceeds by focusing on mice tracks only (Additional file 5: Movie S4).

### The new tracker is highly suitable for studying large-scale dynamics of chemotaxis behavior

To demonstrate the many advantages and the high suitability of the MAT to study complex behaviors, we focused on *C. elegans* nematodes. One of the fascinating complex behaviors that these worms exhibit is chemotaxis, in which animals navigate up a chemical gradient towards the chemical source. Very often, chemotaxis assays are quantified by an attraction index that reflects the end position of the animal, disregarding its intricate trajectories throughout the course of the chemotactic behavior [10]. High-throughput analyses of such trajectories with their relation to the chemical source are imperative to underpinning the mechanisms by which animals navigate through gradients. Our tracking system is particularly suitable for studying complex chemotactic behaviors – it extracts long behavioral tracks of dozens of animals at a time, and positions these trajectories relative to the stimulus source that is set as a reference point.

**Fig. 3:**
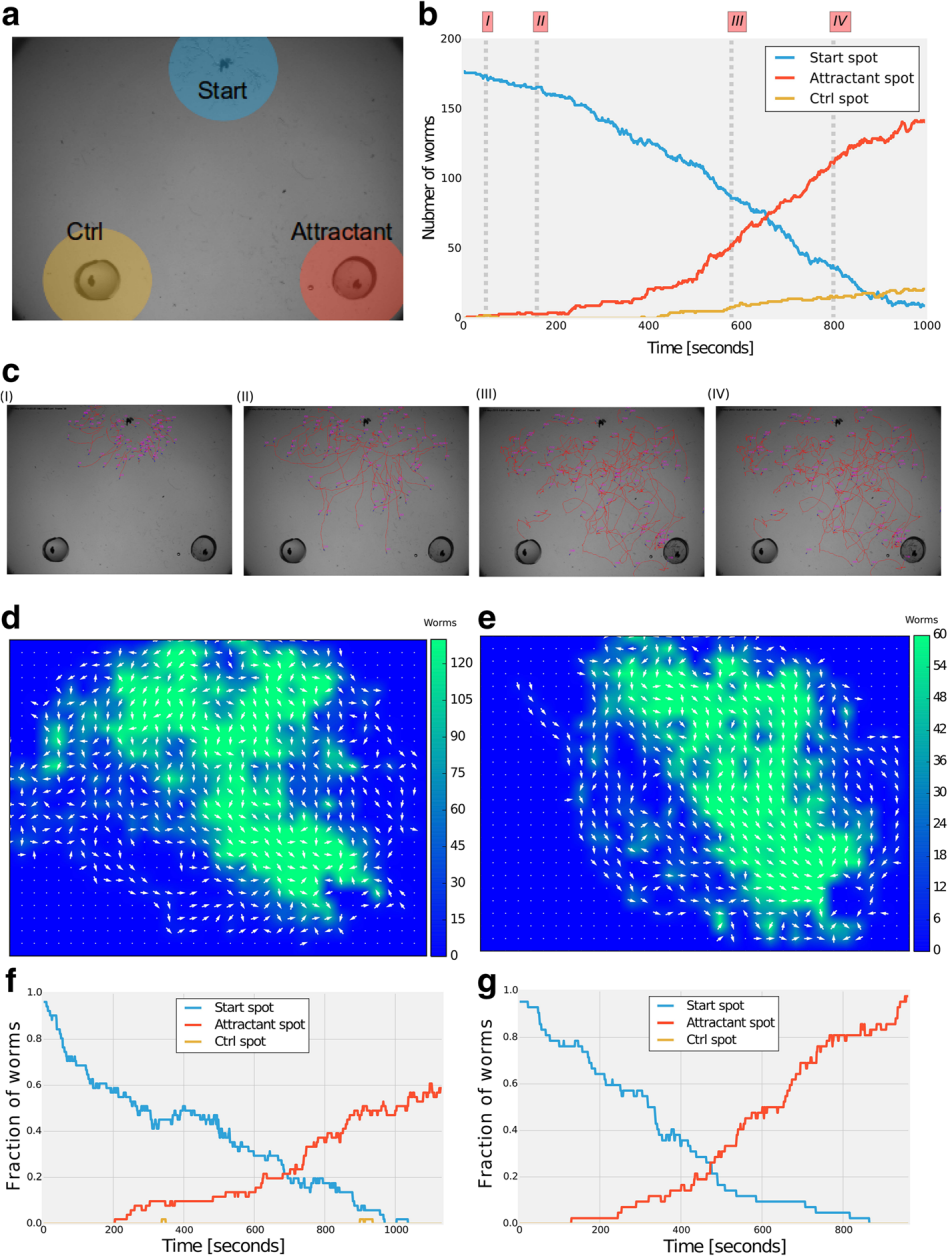
The Multi-Animal Tracker is particularly suitable for studying complex behaviors such as chemotaxis. **a** An image of the experimental chemotaxis plate. Agar plugs soaked with the attractant odorant (red circle), or the control buffer (yellow circle), are placed on the plate’s lid. Neither the attractant, nor the buffer, come in contact with the agar on the plate. Approximately 150 worms are loaded at the starting point (blue circle). The chemoattractant source, the buffer source, and the starting point form an imaginary equilateral triangle with an edge of 4 cm. **b** A quantitative cumulative dynamics of worm position in the chemotaxis assay over the course of approximately 15 minutes. The lines indicate the number of worms in each of the regions of interest throughout the experiment (color coded). In this experiment, nearly 180 worms were loaded on the assay plate. About two-thirds of them reached the chemoattractant during the first 15 minutes of the assay. **c** Images were taken at the specific time points (I–IV) throughout the assay (indicated as dashed lines in **b**) to illustrate chemotaxis progression in the assay plate. Shown are also the trajectories (red) as identified by the tracker software. **d**–**g** The software suite includes a module to generate Attraction Fields (AF), a visualization designed to provide spatial representation of the chemotaxis process throughout the experiment. Shown here are AFs of two chemotaxis assays in which the isoamyl-alcohol attractant was used in 10^−4^ (**d**) and 10^−2^ (**f**) dilutions. The experimental field is binned to squares (35 × 25 in this case, but any binning defined by the user is possible). Arrows represent the average direction of the worms and the color code indicates the overall occupancy throughout the course of the experiment (15 minutes, ~1000 frames). **e**, **g** Chemotaxis dynamics as detailed in section b. Together, these plots provide a full spatiotemporal representation of the chemotaxis behavior of multiple animals over the course of thousands of captured frames

We used the tracking system to extract trajectories of a large number of animals tracked simultaneously during 30 minutes of chemotaxis. To study the chemotaxis performance quantitatively, we defined three regions of interest (ROIs, Fig. 3a), namely the start point where a drop containing over 150 worms is placed (Blue), an area circling the spot of the chemotactic cue (Red), and an area circling the control area typically spotted with the buffer solution used to dilute the chemical cue (Orange). The tracking software counts the number of worms entering or leaving each of these ROIs, thus providing a quick and quantitative analysis of chemotaxis kinetics that can be viewed as a temporal variation of the often used ‘chemotaxis index’ (Fig. 3b, c).

Presenting the rich trajectory data for multiple worms may become cumbersome given the enormous amount of data obtained by the tracking of multiple worms in a spatiotemporal manner over long time periods. To overcome this, we developed the Attraction Field (AF) view, which captures many of the intricate parameters extracted throughout the course of the experiment in a single representation (Fig. 3d, f). For this purpose, we binned the entire field of view into squares (for our resolution we used 35 × 25 squares), and for each square we plotted both the average direction of the worms (arrows) and the overall occupancy over time (color coded). This AF representation provides a quantitative spatial measure of the chemotactic response; for example, higher chemoattractant levels yield more direct trajectories towards the chemoattractant from wider regions in the experimental field and higher worm occupancy around the target region compared to lower levels of the chemoattractant (Fig. 3d–f). When combining the AF views with the ROI kinetics representation (Fig. 3f, g), these data culminate in a rich quantitative spatiotemporal representation of the chemotactic response as measured simultaneously for multiple worms over long time periods. The AF and ROI analyses are supplied as built-in functions in our software analysis suite (Additional file 1).

**Fig. 4:**
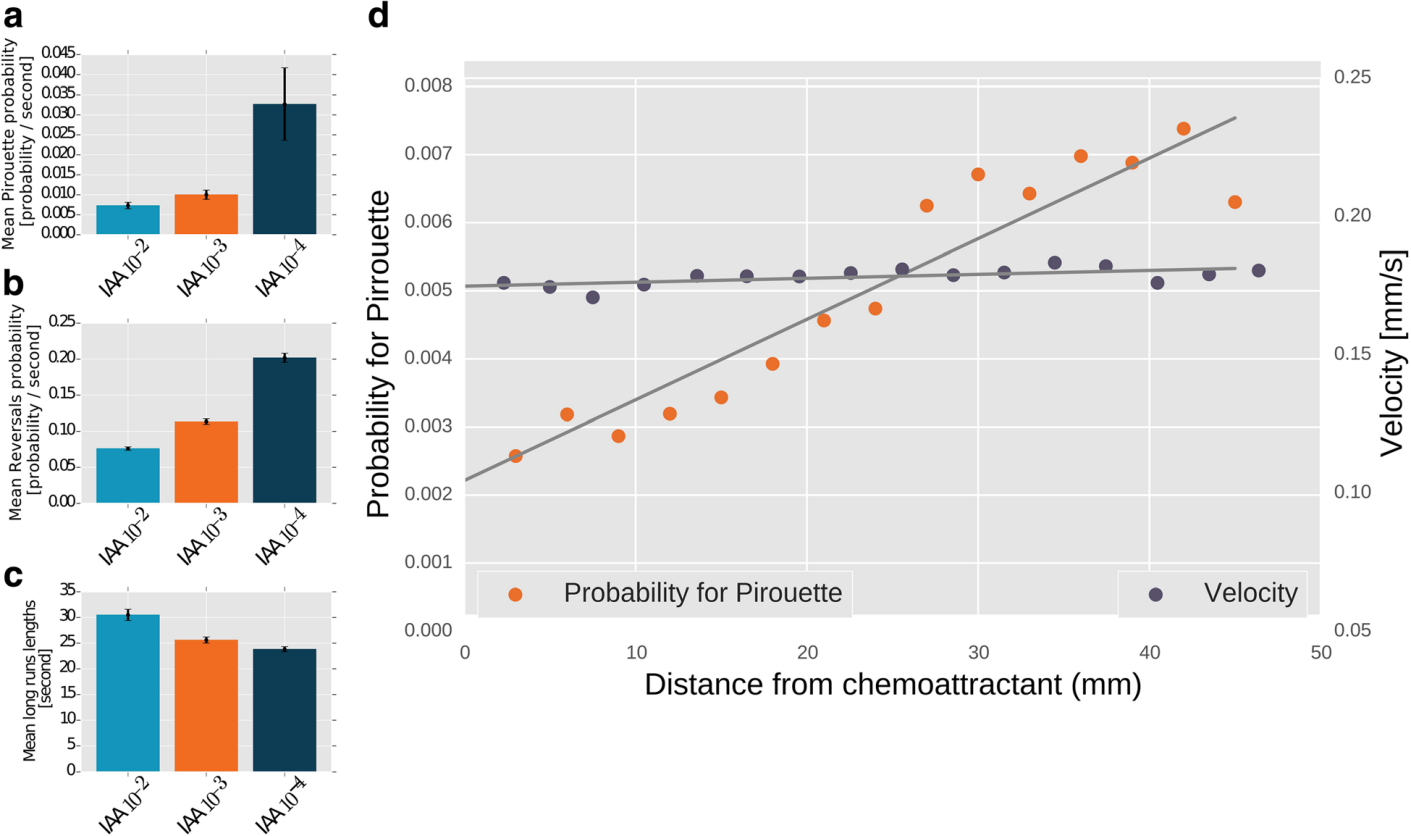
Analysis of chemotaxis parameters. The software extracts several parameters that describe the chemotaxis performance: (**a**) mean probability for a pirouette, (**b**) mean probability for a reversal, and (**c**) mean run lengths (time between reversals). All these parameters are dose dependent – mean probabilities for pirouettes and reversals increase with decreases is the concentration of the attractant. Conversely, mean run length time decreases. Error bars denote SEM of individual tracks. For the different concentrations of isoamyl-alcohol: 10^–2^, we averaged 447 tracks in total; 10^–3^, 1195 tracks in total; 10^–4^, 1470 tracks in total. **d** Probability for a pirouette increases the further the animal is located from the attractant, while the speed remains constant throughout the course of chemotaxis. Points are averages taken from 64 experiments comprising 7505 pirouette events and > 10^5^ speed points

Three key movement features define chemotaxis behavior in worms and presumably in other animals (e.g., fly larvae [25]). These include reversals/sharp turns, runs, and pirouettes that are defined as bouts of multiple sharp turns and reversals [12, 26]. As expected, these three parameters are dose dependent – the higher the chemoattractant concentration, the fewer the pirouettes and reversals and the longer the run times (Fig. 4a–c, Additional file 11: Figure S3). Interestingly, we find that an animal’s speed remains constant throughout the chemotactic behavior and is independent of the distance from the target. Conversely, the probability for a pirouette grows linearly with the distance from the target (Fig. 4d).

### Worms incorporate directional mechanisms to improve chemotaxis efficiency

Surprisingly, the above experiments provided new insights regarding chemotaxis strategies. During chemotaxis, worms use two main strategies, namely (1) a biased random walk (klinokinesis) where worms increase or decrease turning rates depending on whether they crawl down (*dc*/*dt* < 0) or up (*dc*/*dt* > 0) a chemical gradient, respectively [12]; or (2) a weathervane movement (also known as klinotaxis [27]), where worms moving perpendicular to the gradient reorient to move towards the gradient. Interestingly, Shimomura et al. [12] showed that worms’ klinokinesis is not entirely composed of uniform reorientation events, and worms that are off-course just before the pirouette tend to compensate with a larger change in their direction following the pirouette such that they are better oriented towards the source.

**Fig. 5:**
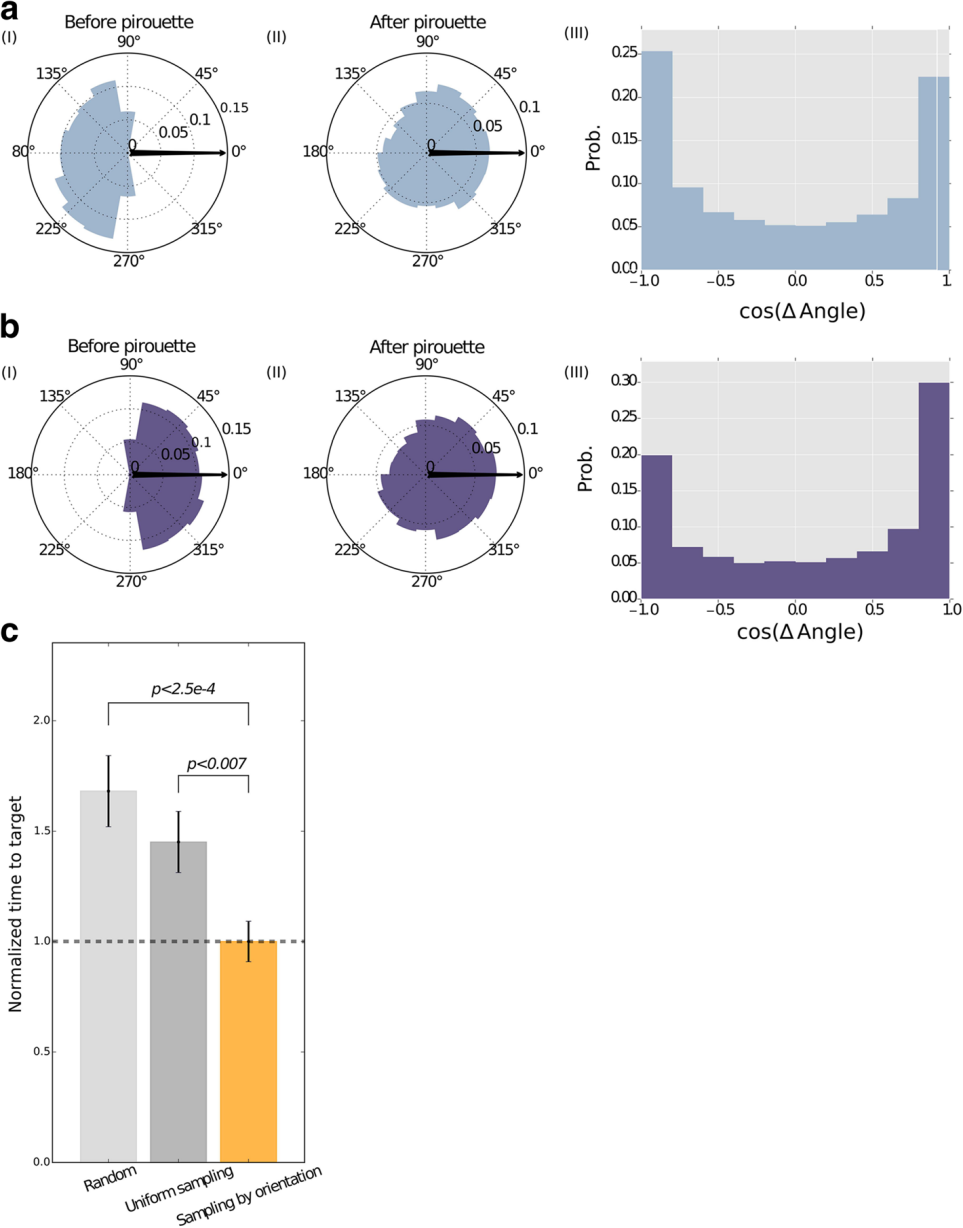
Worms improve their orientation towards the target following the exit from a pirouette. **a** (I) Angular bearing histogram of worms oriented off-course (90° < *B* < 270°) immediately before initiating the pirouettes (*B*_*Before*_); (II) Angular bearing histogram of the worms from (I) immediately after the pirouettes (*B*_*After*_); (III) A histogram of the cosine of the difference in the bearings before and after a pirouette *cos*(*B*_*Before*_ – *B*_*After*_) for worms that were initially off-course. The histogram shows two peaks (around 1 and –1) indicating that worms tend to perform either extreme (e.g., 180°) or minute (e.g., 0°) angle changes. However, the tendency to perform a pirouette with a larger angular difference in bearing is significantly more probable (*P* < 10^–6^, permutation tests, see Methods). The data is composed of 13,297 disoriented pirouette events. **b** (I) Angular histogram of bearings for worms oriented on-course (0° < *B* < 90° or −90° < *B* < 0°) immediately before the initiation of a pirouette (*B*_*Before*_). (II) Angular histogram of worms bearing that are on-course immediately after the pirouette (*B*_*After*_). (III) A histogram of the difference in the bearings before and after a pirouette *cos*(*B*_*Before*_ – *B*_*After*_) for on-course worms. As in off-course worms, these initially on-course oriented worms tend to perform either extreme or minute angle changes, but the frequency of minute changes (*cos*(*Δ* angle) = 1) is significantly higher (*P* < 10^–6^, see Methods). The data is composed of 15,368 oriented pirouette events. **c** Simulations of chemotaxis trajectories. We used three different strategies for choosing the exit angle from a pirouette (see Methods for details). The experimentally observed principle, where the exit angle is sampled according to the entry angle, provides an efficient chemotaxis strategy reflected by the significantly shorter time to reach the target point (*P* < 0.007 and *P* < 2.5 × 10^–3^, Wilcoxon rank-sum test for random and uniform sampling, respectively). Error bars denote SEM of the number of simulated worms in each simulation. Overall, we simulated 250 worms per strategy

Our experimental results not only recapitulated these observations (Fig. 5a), but also provided novel understanding of this complex behavior; worms, originally directed towards the chemoattractant, tend to maintain their general direction following a pirouette (Fig. 5b, see also Methods). This, in addition to the observations made by Shimomura et al. [12], explains why both directed (–90° < B_Before_ < +90°) and undirected (+90° < B_Before_ < 270°) trajectories improve their general direction following a pirouette (Fig. 5aII, bII; Rayleigh Z-test, *P* < 10^–5^, for both directed and undirected). Directional changes that follow pirouette events show a bimodal distribution where small (ΔB ≈ 0° rad, cos(ΔB) ≈ 1) and large (ΔB ≈ ± 180° rad, cos(ΔB) ≈ –1) changes make nearly half of the total directional changes (Fig. 5aIII, bIII).

To understand the functional significance of this reorientation strategy we simulated chemotaxis of worms adopting different criteria for choosing directional changes following a pirouette (sampled from our experimental data, see Methods for details). Interestingly, chemotaxis performance of worms simulated according to the strategy detailed above was superior over other possible strategies as the worms reached the target significantly faster (by approximately 50%, *P* < 10^–5^, Wilcoxon rank-sum test, Fig. 5c). These findings provide a novel understanding of animal chemotaxis strategies, and further underscore the significant roles of pirouettes in mediating efficient chemotaxis.

### Worms integrate environmental cues and enhance chemotaxis towards richer environments

Integrating information extracted from the environment is an important ability shared by many animals [28, 29]. In particular, during food search, animals often face confusing cues and attending to the most reliable of them is likely to improve their chance of quickly reaching food sources [30]. For example, multiple cues originating from a source may be rendered as a more reliable food source than a source emitting a single cue.

**Fig. 6:**
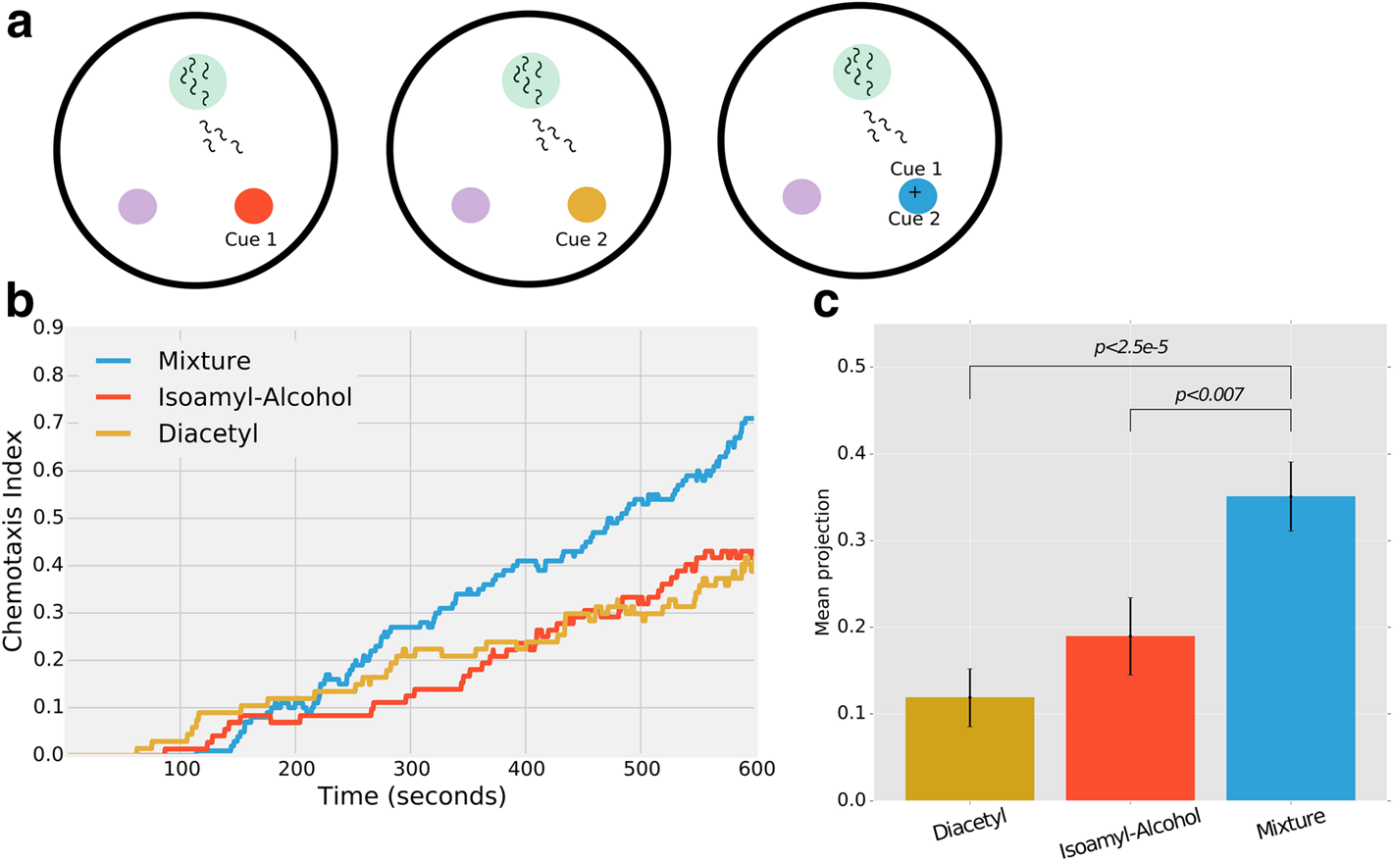
Worms integrate environmental information preferring richer environments. **a** Integration experiments were performed by comparing worms’ chemotaxis in three different assay plates. Two of the plates contained only one of the two cues each (e.g., diacetyl (DA) and isoamyl-alcohol (IAA)). The concentrations for the two cues were predetermined via dose-response assays and equipotential concentrations (i.e., attracting the worms to the same extent) were chosen. A third assay plate contained a mixture of both cues, each cue in a concentration that is half of what was used in the single cue assay plate. **b** Chemotaxis dynamics for the three assays. In the single chemoattractant experiments, we used a volumetric concentration of 0.75 × 10^− 5^ for DA, and 0.5 × 10^− 4^ for IAA. Accordingly, the mixture was composed of 0.37 × 10^− 5^ of DA and 0.25 × 10^− 4^ of IAA. We fitted a linear curve to the dynamic curves and estimated the linear coefficient (*β*1) to describe the potential of the cues to attract animals (*DA*: *β*1 = 0.5 ± 3.3 × 10^–3^; *IAA*: *β*1 = 0.5 ± 2.1 × 10^–3^). Chemotaxis towards each of the individual cues was very similar (as pre-calibrated by dose-response assays) indicating their equipotential attraction. The mixture, however, was significantly more attractive than each of cues alone. Shown is an example of a single experiment where approximately 100 worms were loaded on each plate. **c** The mean projection, the component of the movement towards the target, is significantly higher in the mixture compared to each of the cues alone (*P* < 2.5 × 10^–5^ and *P* < 0.007, compared to DA and IAA alone, respectively). Error bars denote SEM of all tracks together taken in a specific region of the experimental field. These data are composed of 92 run bouts for DA; 96  run bouts for IAA; and 159  run bouts for the mixture

We employed our MAT system and asked whether *C. elegans* can integrate environmental information (i.e., attend to more than one cue at a time) presented as food odorant cues. To do this, we compared worm chemotaxis towards each of two cues, isoamyl-alcohol (IAA) and diacetyl (DA), when presented independently and when combined as a single mixture (Fig. 6a). Specifically, we used the same experimental design as detailed above for the chemotaxis assays (Figs. 3, 4, and 5). We first assayed worm chemotaxis to each cue in a dose-dependent manner and chose two equipotential concentrations (the concentration of each cue that attracts the worms to the same extent, see Methods). Both stimuli at these concentrations attract worms with the same kinetics (*DA*: *β*1 = 0.5 ± 3.3 × 10^−3^; *IAA*: *β*1 = 0.5 ± 2.1 × 10^−3^; *β*1 denotes the coefficient of the liner regression of chemotaxis dynamics, Fig. 6b). Interestingly, worms are significantly more attracted when these cues are combined and presented as a mixture, as many more worms reach the target area with faster kinetics (*Mixture*: *β*1 = 0.94 ± 4.1 × 10^−3^, Fig. 6b). Importantly, we halved the concentration of each stimulus in the mixture compared to its concentration when presented individually, and yet worms were significantly more attracted to the combination of the cues (*P* < 0.007 compared to IAA and *P* < 2.5 × 10^−5^ compared to DA, Fig. 6b). This suggests that worms can attend both cues concomitantly as they navigate in search of food.

We next asked which features in the chemotaxis behavior underlie this enhanced attraction. For this, we analyzed the directness of the worms towards the different stimuli by quantifying the projection of their steps on the vector that connects the worm and the chemoattractant (see Methods). We found that worms presented with a mixture of cues move more directly towards the source than worms presented with each cue alone (*P* < 0.006, Fig. 6c). Together, these data demonstrate that worms can integrate environmental information and attend to more than one cue at a time. Furthermore, this integration is translated to behavioral outputs manifested primarily by the ability to better reorient the trajectory following a pirouette (Additional file 12: Figure S4).

### Quantitative analyses of worm motility during aging and neurodegenerative-linked proteotoxicity progression

Finally, we demonstrate the powerful advantages of using this novel MAT in studying fine locomotion deficits associated with aging and neurodegenerative-related diseases. During these processes, animal locomotion deteriorates, culminating eventually in complete paralysis [31, 32]. It therefore becomes extremely challenging to quantify minute locomotion changes during these critical periods. Moreover, due to large locomotion variability within worm populations it is critical to collect large data sets in order to obtain reliable and reproducible results [33]. The MAT system overcomes these hurdles owing to its high-throughput and accurate tracking.

**Fig. 7:**
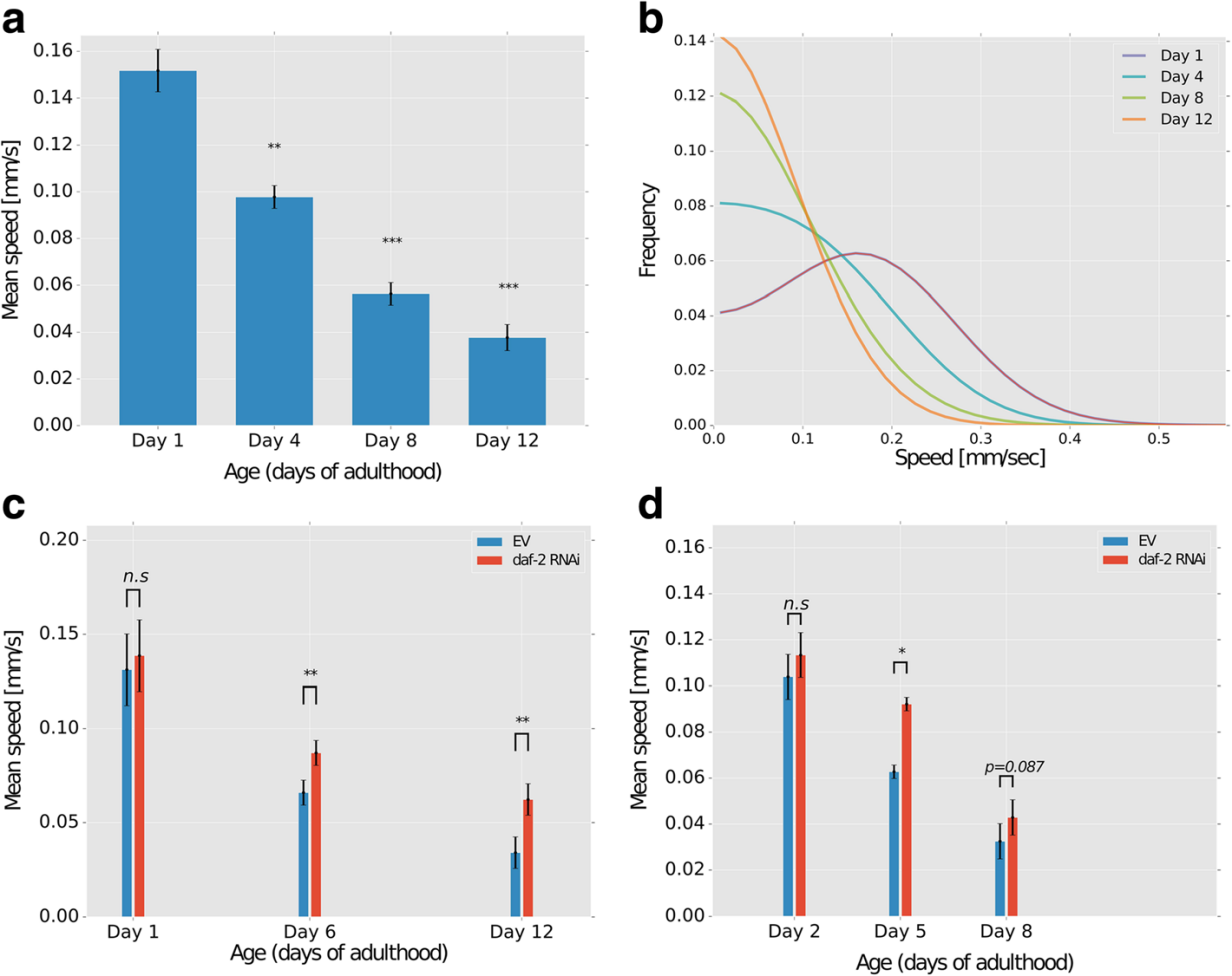
The Multi-Animal Tracker (MAT) is compatible with accurate high-throughput measurements of aging- and neurodegenerative-associated locomotion decline. **a** Synchronized CF512 worms were grown to 1, 4, 8, and 12 days of adulthood. An agar plug soaked with 1 μL of the repellent 2-nonanone (1:10 diluted in ethanol) was attached to the plate lid and worm movement was recorded 2 minutes thereafter for 1 minute. Analysis using the MAT showed constant aging-associated decline in mean speed. **b** Speed distributions within worm populations were unimodal, indicating that the mean value was not biased by a subpopulation of paralyzed or dead worms. The number of speed points in both (**a**) and (**b**) that we averaged on were 30,512 for Day 1; 23,672 for Day 4; 9401 for Day 8; and 6240 for day 12. **c** CF512 worms were grown to days 1, 6, and 12 of adulthood on either control bacteria (EV) or on *daf-2* RNAi bacteria. Repellent was added and worm movement was analyzed as in (**a**). Analysis using the MAT confirmed that reduced insulin/IGF-1 signaling (IIS) slows age-associated motility decline. **d** AM140 worms expressing the aggregative polyQ35-YFP fusion protein were grown to days 2, 5, and 8 of adulthood on either control EV or *daf-2* RNAi bacteria, and speed was scored as in (**a**). Note that x-axis is drawn to scale with panel **c**. Speed declines as worms age and proteotoxicity progresses, but reduced IIS slows motility decline. Error bars denote SEM of three biological repeats for panel a and four biological repeats for panels **c** and **d**. Each biological repeat consists of 2–3 replicate plates with dozens of worms per plate. Detailed data points are provided in Additional file 13: Table S1.* *P* < 0.05; ** *P* < 0.005; *** *P* < 0.0005

To quantify locomotion during aging, we used synchronized worm populations and measured animal motility as they age. As expected, we found a steady and significant decline in animal motility that positively correlated with age (Fig. 7a, b). Moreover, as velocity is considerably low in aged animals, our tracker faithfully identifies multiple worms at a time to score minimal displacement events at the single pixels per second resolution. Importantly, the significant decline in the average animal speed was not due to possible death of a fraction of the animals (for which we may score a zero speed). To rule out this possibility, we set our tracking algorithm to discard worm events that showed no activity throughout the experiment. This ensured that dead animals would not skew and bias the experimental results.

We next employed our tracker to study aging-associated motility impairment following external manipulations such as those used in large-scale RNAi or chemical screens. In *C. elegans*, knocking down the sole insulin/IGF-1 signaling (IIS) receptor, *daf-2*, leads to long-lived, stress-resistant animals that maintain robust proteostasis [34–36]. Indeed, worms treated with *daf-2* RNAi showed a significantly slowed deterioration in motility starting from day 6 and on (Fig. 7c). Similarly, we utilized our MAT system to quantitatively measure proteotoxicity-associated locomotion decline. For this, we tracked motility of worms expressing the aggregative peptide composed of 35 glutamine repeats (polyQ35) in their body wall muscles, which leads to motility impairment in a time-dependent manner [37]. As velocity declined with age, animals treated with *daf-2* RNAi exhibited a significantly slower decline, indicating the protective role of inhibiting IIS in proteotoxicity (Fig. 7d). In particular, the MAT system reliably detected minute motility differences (fractions of mm/sec) that ultimately reflect significant differential aging and proteotoxicity paces. Together, these results demonstrate that our MAT system is highly suitable for studying proteotoxicity- and age-related maladies in large worm populations, enabling fast, high-throughput, and accurate screens of mutant animals or RNAi libraries.

## Discussion

Herein, we present a novel MAT that allows the tracking of multiple animals simultaneously over long time periods. Importantly, this user-friendly system offers an end-to-end solution – from acquisition and recording movies, through tracking algorithms and advanced built-in analyses functions, to displaying the rich data in comprehensive and informative plots.

A major advantage of our system over existing multi-worm trackers is that we implemented a machine learning algorithm coupled with a Kalman-type predictor [24]. These features turn the tracker into a multi-purpose system compatible with tracking various animals differing in size, appearance, velocities, and other activity parameters. Indeed, we show that the software can faithfully track nematodes, flies, zebrafish, and mice (Fig. 1, Additional file 2: Movie S1, Additional file 3: Movie S2, Additional file 4: Movie S3, Additional file 5: Movie S4). Moreover, the tracker is indifferent to the acquisition systems (e.g., a wide variety of optical setups can be used), and it does not require adjusting parameters for particular environments or experimental settings (e.g., resolution, brightness, hue, etc.).

Combining machine learning algorithms together with the predictor also supports accurate detection and segmentation of animal entities, specifically so when background environments are ‘noisy’. For example, long temporal experiments are challenging as the environment is dynamic and often changes over the course of the experiment (e.g., water condensation on the plate lid). Our system reaches approximately 90% detection accuracy (precision and recall) when tracking around 100 worms simultaneously (Fig. 2 and Additional file 7: Figure S2). Furthermore, high-throughput studies that track multiple animals at a time pose an additional challenge – the greater the number of animals tracked, the higher the chances that they will collide, therefore precluding the possibility to track individuals over long time periods. However, implementing the predictor in our system significantly aids in resolving such collision events (Fig. 2c and Additional file 7: Figure S2).

Machine learning approaches had been previously applied to tracking single worms (for example, see [38, 39]). Our approach, however, is slightly different and is particular suitable for tracking multiple animals in long-temporal experiments in which noise accumulates over time. We allow the user to first set a fixed thresholding parameter (as part of the training process), and then the user chooses segmented entities corresponding to genuine animals. Based on the features of these entities, a classifier is learned and is subsequently used to classify other segmented entities throughout the movie. Indeed, using several clicks on animal entities, the user ‘trains’ the algorithm to tell apart genuine animal entities from possible background noise, thereby significantly reducing erroneously detected animals. Importantly, this learning step typically takes 1 minute, after which the algorithm automatically extracts animal entities from thousands of frames. If the same settings are used for subsequent experiments then the same training set can be used for their analyses. Together, the machine learning algorithm and the predictor, the broad possible usage, and the ability to simultaneously track multiple animals over long time periods make this MAT into a unique and powerful system for studying animal behavior.

Various other machine-vision algorithms that support high-throughput tracking of many individual animals simultaneously are also available (i.e., in flies [40]). Similarly to our tracker, these systems automatically track large groups of unmarked animals. Furthermore, machine learning methods were developed to support automatic behavioral classifiers based on animal trajectories alone, which subsequently infer higher order behavioral outputs such as social and individual behavior [41]. While our tracking system, together with its built-in analytic functions, is primarily geared to studying complex spatial navigation, it can also be used to study other complex behaviors. It will therefore be interesting to combine such machine learning post-acquisition methods to extend the repertoire of complex behaviors to be studied.

Interestingly, our initial experience with the MAT system already yielded novel insights into the intricate chemotaxis behavior. In a seminal study, Shimomura et al. [12] demonstrated the critical role of pirouettes (a bout of successive turns) in chemotaxis, where worms entering a pirouette with an off-course angle tend to exit the pirouette better oriented towards the center of the gradient. Our tracking analyses confirmed these results and added an additional important piece to this fascinating strategy – worms that enter the pirouette on-course oriented towards the target tend to maintain this on-course trajectory following the pirouette. Together, these strategies greatly enhance an animal’s navigation abilities, enabling it to reach a target source significantly faster (as we also corroborated through simulations, Fig. 5c).

In addition, we used our MAT system to show that *C. elegans* animals can integrate environmental cues (Fig. 6). Such integration is an important feature to predict food abundance and quality [13]. Interestingly, we found that this sensory integration is manifested in the directional changes in the pirouette events – animals crawling towards a richer target are more likely to exit the pirouette at an angle that is more directed towards the target (Additional file 12: Figure S4). In our experiment, we used two cues, DA and IAA, sensed primarily by AWA and AWC sensory neurons [42–45]. These two sensory neurons synapse on mutual downstream interneurons (AIA, AIY) that, along with additional inter and command neurons, control worm locomotion [46–48], thus presumably serving the point where sensory information is integrated before dictating locomotion outputs.

We also demonstrated that the MAT system allows the study of aging and proteotoxicity-associated diseases. These processes are characterized by gradual locomotion decline, and quantitative accurate measurements are required to study this slow progression. Our system accurately captures such minute locomotion changes (Fig. 7). Moreover, aging and proteotoxicity effects widely vary from animal to animal, requiring assaying large population cohorts. The novel MAT system is particularly suitable to this end as it allows accurate tracking of animal locomotion in a high-throughput manner, and can therefore serve as a powerful system for extensive laborious screens of mutants or chemical and RNAi libraries.

## Conclusions

Herein, we present a novel MAT that provides accurate high-throughput analyses of complex behaviors. Importantly, this user-friendly system is easy to operate and does not require prior programming skills. With a wide range of possible uses and the compatibility of studying various animal models, this MAT will serve as an important system for elucidating novel principles underlying complex behaviors.

## Methods

### The MAT

The software suite, together with its freely-available code, can be downloaded through github (https://github.com/itskov/MultiAnimalTrackerSuite). The downloaded files also include a detailed user manual (with exemplar analyzed movies) for the installation and use of the different packages in the software.

### Machine learning algorithm for the identification of behaving animals

#### Segmentation and feature selection

To minimize tracking of erroneous objects, we utilized a supervised machine learning approach. Prior to tracking, the GUI prompts the user to mark few samples of genuine animal entities (these will form the training set). The GUI presents the user with a frame from the experiment video next to the same frame filtered with the Laplacian of Gaussian (LoG) filter [49, 50]. That is, the image is convolved with a matrix *M*_*LoG*_. In this filter, the standard deviation of the Gaussian, *σ*_*LoG*_, is used to control the preferred size of the entities to be highlighted in the image. Thus, the user is presented with a scroll bar with which it can control the size of the *σ*_*LoG*_. Basing the filter on the standard deviation of the Gaussian, *σ*_*LoG*_, allows the system to mark and track entities drawn from different imaging settings, and/or different model organisms. The chosen *σ*_*LoG*_ are subsequently used to segment the entities that will be then classified as ‘animals’, or else considered as background, and thus discarded.

Next, the tracker extracts features from the entities selected by the user. We designed the tracker to extract the smallest subset of features to enable good performance with a wide range of different model organisms and imaging settings. These features include the area of the entity, and the mean, median, minimum, and maximum of the entity's pixels intensity. While this small set of general features is sufficient to accurately segment animal entities (all the results presented within this paper use these features only), additional features may be included at the users discretion, which will support further flexibility and efficient segmentation under various circumstances. The open source code can be easily accessed and modified as necessary; the identity of the extracted features is found in the ‘extractFeatures’ method, which is in the tracker object.

#### The algorithm for calling or rejecting animal entities

Since users choose only ‘positive’ entities, and they are not asked to mark entities which are not animals, a traditional machine learning approach (as a linear separator, decision tree, etc.) could not be used. Alternatively, we found the following described method to be superior to regression-based classification methods:

Denoting *m* as the number of entities marked by the user, and *n* as the number of features extracted by the tracker, then *S* is a matrix of size *m × n*, for which, for any *i,j* entry, the value of *S*_*ij*_ is the value of the *j* ^*th*^ feature of the *i* ^*th*^ marked entity. We also denote Σ (of size *n × n*) as the covariance matrix of the different features as estimated from *S*. We measured the distance *D* of each sample in the training set, *S*_*i*_, from the estimated distribution of the features in the video, defined by Σ and *μ*_*S*_ *= E(S)*, using the Mahalanobis Distance [51]:

$$ D\left({S}_i\right)=\sqrt{\left({S}_i-{\mu}_s\right) T\cdot {\sum}^{-1}\cdot \left({S}_i-{\mu}_s\right)} $$

We observed that the distribution of the distances calculated as above appeared to be unimodal. Thus, we formulated a rejection rule for new entities based on the distribution of the Mahalanobis distances of samples in the training set. Any new entity *x*, an *n × 1* vector, was classified as an ‘animal’ if it held that,

$$ D(x)\le T\left(\beta \right) $$

Where *T*(β) is the value of the Mahalanobis distance, which is larger than (1 – β) × *m* samples in the training set. Throughout all our experiments, we set β to be 0.96; however, this value can be easily adjusted to control type I and type II errors in the classification.

#### Tracking algorithm

We used a simple tracking algorithm which matches spatially closest entities between consecutive frames (the only entities considered are entities that were labeled as animals in the previous step). We incorporated a simple variation of the Kalman filter [24] to predict the position of animals that are obscured by other entities as, for example, in the case of collisions or in ambiguity  in matching entities between consecutive frames. Prior to matching an active track to a new entity found in a new frame, the tracker calculates the track's predicted position based on the animal's recent velocities and accelerations. In case the tracker fails to match a single entity to an active track, it will match the track to its predicted position. The tracker will maintain tracking when the animal reappears, or will discard its prediction if no animal entity was found to match the track during the three subsequent frames.

#### Evaluating tracker accuracy

We performed several analyses to evaluate tracker accuracy. First, we generated a ‘gold standard’ set of approximately 500 worms that were manually identified and marked. Using this training set (which reflects the presumably optimal human-eye tracker), we could estimate the precision of our segmentation and worm identification process. Specifically, we calculated three well-known parameters in the field of object identification, namely Precision, Recall and Fscore, all as a function of different training sizes. These parameters, provided in Fig. 2b, are defined as follows:

(I) Precision: out of X entities classified as worms by the software, how many were actual worms? Based on the machine learning algorithm alone, we find this measure to reach nearly 90% accuracy. In practice, the prediction feature adds on top of this to gain over 90% precision.
(II) Recall: out of all worms genuinely found on the plate (based on the ‘gold standard’ set), how many are indeed classified by the tracker as worms? Figure 2b shows that the tracker reaches over 85% accuracy even when using only 50 worms for the training set.
(III) Fscore is the harmonic average of the two parameters (Precision and Recall).

In addition, we assessed the tracker performance when disabling the machine learning and the Kalman-type predictor. We find that enabling these features significantly improves animal segmentation and tracking (Fig. 2c and Additional file 6: Figure S1 and Additional file 7: Figure S2). In particular, these features are important even when analyzing movies that initially seem to be ‘easy-to-analyze’ with high contrast between animals and background (see analyses in Additional file 7: Figure S2 and Additional file 8: Movie S5, Additional file 9: Movie S6, Additional file 10: Movie S7).

##### Evaluating worm resolution following collisions

To estimate the precision by which our system correctly resolves worms after collision, we have taken a similar approach as described above to evaluate tracker accuracy. We first defined collisions as events in which worms have come to a close proximity (less than 15 pixels which correspond to approximately 0.4 mm in our setup), and manually matched worm identity before and after the collision event. This is to be used as our ‘gold standard’ measure. We then ran our tracker in two modes, with and without the predictor. The results of the accuracy of predicting the correct tracks following collisions are summarized in Additional file 6: Figure S1b. As evident, applying the predictor significantly improved detection of worms after collision as only approximately 2% of the cases were not resolved (Resolved 0), whereas without implementing our predictor, 46% of the collisions were not resolved. Furthermore, in 44% of the collision cases, one worm was correctly resolved (as opposed to 16% without the predictor) and in 55% of the cases both worms were correctly resolved (as opposed to 38% without the predictor).

##### Chemotaxis assays

To get a large synchronized population of young adult animals (N2, WT strain), we bleached gravid worms and plated approximately 1000 eggs on a 90-mm standard NGM plate pre-seeded with 500 μL *E. coli* OP 50 culture. These worms were assayed 3 days later when they reached young adulthood (YA). Before the experiment, the YA worms were rinsed off the growth plates and washed three times in chemotaxis buffer (1 mM CaCl_2_, 1 mM MgSO_4_, 5 mM K_3_O_4_P, pH 6.0). Chemotaxis assays were performed on Chemotaxis plates, which include the same ingredients as the chemotaxis buffer with the addition of 2% agar. Importantly, worms were grown at 20 °C and behavioral assays were also performed in a temperature-controlled room at 20 °C.

We marked an equilateral triangle on the plate’s lid (90-mm round plates) with an edge length of 3 cm. We used agar chunks soaked (15 μL) with the chemoattractants of choice and placed them on two of the triangle vertices (Fig. 3a). On the third vertex, we placed a 5-μL Chemotaxis Buffer drop of washed worms (we first estimated worms’ concentration in the pellet following the last wash to plate a desired number of worms). Chemotaxis assays were then imaged using a Photometrics Micropublisher 5 MB camera, using Olympus SZ61 binocular equipped with a 0.5× lens. To acquire movies we used our own in-house imaging software that is freely available with this MAT and which uses MATLAB’s image acquisition toolbox. Movies were acquired at a rate of one frame per second.

##### Calculating worms’ directness in relation to the chemoattractant target

We quantified worm’s directness towards the chemoattractant at time *t* by obtaining the projection of the worm’s velocity (*v*(*t*)) on the vector that connects the worm and the chemoattractant (*d*(*t*), normalized). That is, for each step of each worm, we calculated the following dot product:

$$ proj(t)=\frac{< v(t), d(t)>}{\left| d(t)\right|} $$

In the integration experiments we used only projections of the worms that were on their way towards the chemoattractant for the first time (and ignored revisiting worms).

##### Identification and analyses of pirouettes

To study the role of pirouettes during chemotaxis, we used the same notations as described previously by Shimomura et al. [12]; we defined *bearing* (B) as the angle between the worm’s velocity vector and the spatial vector between the worm’s position and the peak of the chemoattractant. We used B_Before_ and B_After_ as the bearing immediately before and immediately after a pirouette event, respectively, and ΔB as B_Before_ – B_After_.

We defined sharp turns as succeeding movement vectors with an angle of > 100° between them, and used the definition suggested by Shimomura et al. [12] for a pirouette, which is a bout of sharp turns. Following the observation that run distribution can be described by the sum of two exponents [12], we chose the minimal size of a pirouette to be T_crit_ = 5 sec. Any run shorter than this will be considered to be a component of a pirouette. We defined B_before_ as the average bearing for three consecutive steps prior to the pirouette, and similarly we defined B_after_ to be the average bearing of three consecutive steps after the pirouette.

##### Integration of environmental cues

We first looked for two chemoattractants that attract the worm in the same manner. To do so, we performed an array of chemotaxis experiments with varying chemicals and concentrations and examined motility parameters such as chemotaxis index dynamics, probability for a pirouette, and lengths of runs, etc. We chose two chemoattractants that showed the same effect on the worm’s chemotaxis: 0.75 × 10^−5^ DA, and 0.5 × 10^−4^ IAA. We then created a mixture of the two chemicals such that each chemical was diluted twice in the final solution, and performed the chemotaxis assay with it. We compared chemotaxis dynamics, directness, and bearing between the single chemoattractant experiments and the mixture.

##### Chemotaxis simulations

To study the significance of directional changes following a pirouette in light of our findings, we simulated worm courses towards an attractant using three different reorientation strategies, namely (1) choosing the directional change (ΔB) uniformly between –180° and +180°; (2) uniformly sampling from the directional changes observed in our experiments regardless of the specific angle in which the worm entered the pirouette; and (3) we first divided the directional changes observed in our experiments into two groups – one group contained directional changes made by worms which were initially directed towards the target (–90° < B_Before_ < +90°), and the other contained directional changes made by off-course worms (+90° < B_Before_ < 270°). We next chose the directional change based on the angle of the ‘simulated worm’ just prior to the pirouette (e.g., if the worm was initially directed towards the target then the directional change was sampled from the directional change group of the directed worms). Interestingly, worms simulated using the third strategy reached their target significantly faster than if simulated using the first two strategies (Fig. 5c).

For these simulations, we used a simple model considering a minimal number of parameters, namely worm start point, chemoattractant position (target point), and probabilities for a pirouette of directed and undirected worms. The first two parameters, the start and target coordinates, were extracted directly from the chemotaxis experiments. The probability for a pirouette of directed worms was set to 0.03 per second, reflecting two pirouettes per minute, and the probability for a pirouette of undirected worms was set to be five times more probable (0.15). All the angular differences before and after a pirouette were directly drawn from our experimental data based on directed and undirected worm behavior. For this, we extracted from the experimental data all pirouette instances and calculated angular differences before and after a pirouette and constructed a distribution curve. For simulations, we drew angular angle differences based on these distributions. Importantly, the simulation results were not sensitive to small changes in the parameters set.

### Aging- and neurodegenerative-associated locomotion decline

To study aging- and neurodegenerative-associated locomotion decline we prepared animals as described for the chemotaxis assays above. Briefly, for aging assays, we employed temperature-sensitive sterile worms (strain CF512) that become sterile when exposed to 25 °C during development. We synchronized animals by bleaching gravid animals and performed the motility assays on days 1, 4, 8, and 12 of adulthood. For the *daf-2* RNAi experiments we used empty vector as control, and age-synchronized worm locomotion was measured at days 1, 6, and 12 of adulthood.

To assay locomotion in neurodegenerative-associated disease we used the AM140 strain. These worms express the aggregative peptide composed of 35 repeats of glutamine fused to the yellow fluorescent protein in the body wall muscles [37]. Motility was assayed at days 2, 5, and 8 of adulthood. In all these assays, the repellent 2-nonanone was added to stimulate motility. We started measuring worm locomotion 2 minutes after the addition of 2-nonanone for the duration of 1 minute.

## Additional files

Additional file 1: The Zaslaver’s lab Multi-Animal Tracker (MAT) - a user manual. (PDF 863 kb)

Additional file 6: Figure S1. The tracker implements a machine learning algorithm and a predictor that contribute to accurate detection and precise tracking following collision. (**a**) In a typical long behavioral assay, condensation on the lid and trail marks left behind significantly contribute to entities discovered by the tracker. The number of these entities increases with time (red curve), but since the tracker employs a machine learning algorithm for animal identification, it ignores these erroneously detected entities, and the number of genuine animal entities remains constant throughout the experiment (blue curve). (**b**) The software implements a predictor that aids resolving animals following collisions. The colors/numbers correspond to the number of animals resolved following collision of two animals: 2 – both animals resolved; 1 – only one animal resolved; 0 – none. (**c**) An example of six fully-extracted tracks of six individual worms during 10 minutes of tracking. Overall, approximately 100 worms were loaded on the plate, and the tracker software can often keep track of individual animals despite frequent collisions. (TIF 1660 kb)

Additional file 7: Figure S2. Employing both the machine learning (ML) algorithm and the Kalman-type predictor significantly improves accurate animal detection and tracking. This is true even when analyzing a fairly easy movie with clear contrast between animals and background. We analyzed a crowded arena of swimming zebrafish in three different ways: (**a**) disabling both ML segmentation and the predictor; (**b**) applying ML segmentation but disabling the predictor; and (**c**) applying both ML segmentation and the predictor in the segmentation and tracking algorithm. To provide a quantitative measure for the advantage using both the ML and the predictor we calculated tracks’ length (in seconds) as extracted throughout the movie. Each point (*i,j*) in the heat map represents the average track length between time *i* (y-axis) and time *j* (x-axis). We then identified the segments with the longest tracks in each of these three-way analyses, and averaged them: (a) 0.6 ± 0.02 [s]; (b) 1.15 ± 0.14 [s]; (c) 2.2 ± 0.3 [s]. Thus, employing both the ML and the predictor improves track length by more than three-fold. The movies corresponding to these three-way analyses are provided as Additional file 8: Movie S5, Additional file 9: Movie S6, and Additional file 10: Movie S7, corresponding to (a), (b), and (c), respectively. Note, in Additional file 8: Movie S5 (neither ML nor predictor), the many background entities that are being tracked and the tiny insect that was crawling on the left, which would have been part of the statistics unless ML was used. (TIF 979 kb)

Additional file 11: Figure S3. Various chemotaxis parameters are dose dependent. Histograms of (**a**) probability for a pirouette, (**b**) probability for reversal/sharp-turn, (**c**) run lengths (time between reversals). (TIF 670 kb)

Additional file 12: Figure S4. Worms integrate environmental cues and enhance chemotaxis towards richer environments. When presented with a mixture of cues (**c**, **d**), worms are more likely to maintain their general direction following a pirouette (ΔB < *Π*/2), as compared to worms presented with each of the components separately (**a**, **b**, **d**; *P* < 0.005). We could not detect a significant increase in the probability for a larger directional change for worms that were presented with a mixture, compared to worms that were presented with single components of the mixture (*P* > 0.2). (TIF 943 kb)

Additional file 13: Table S1. The raw data of the individual replicates used to produce Fig. 7a, c, d. (XLSX 208 kb)

## References

[1] Tinbergen N (2005). On aims and methods of Ethology. Anim Biol.

[2] Anderson DJ, Perona P (2014). Toward a science of computational ethology. Neuron.

[3] Brenner S (1974). The genetics of Caenorhabditis elegans. Genetics.

[4] White JG, Southgate E, Thomson JN, Brenner S (1986). The structure of the nervous system of the nematode Caenorhabditis elegans. Philos Trans R Soc Lond B Biol Sci.

[5] Barr MM, Garcia LR. Male mating behavior. WormBook. 2006. The C. elegans Research Community. doi:10.1895/wormbook.1.7.1. 10.1895/wormbook.1.78.1 PMC4780960 18050467

[6] Flavell SW, Pokala N, Macosko EZ, Albrecht DR, Larsch J, Bargmann CI (2013). Serotonin and the neuropeptide PDF initiate and extend opposing behavioral states in C. elegans. Cell.

[7] Raizen DM, Zimmerman JE, Maycock MH, Ta UD, You YJ, Sundaram MV, Pack AI (2008). Lethargus is a Caenorhabditis elegans sleep-like state. Nature.

[8] Mori I (1999). Genetics of chemotaxis and thermotaxis in the nematode Caenorhabditis elegans. Annu Rev Genet.

[9] Vidal-Gadea A, Ward K, Beron C, Ghorashian N, Gokce S, Russell J, Truong N, Parikh A, Gadea O, Ben-Yakar A, et al. Magnetosensitive neurons mediate geomagnetic orientation in Caenorhabditis elegans. Elife. 2015;4. doi:10.7554/eLife.07493. 10.7554/eLife.07493 PMC4525075 26083711

[10] Bargmann CI, Horvitz HR (1991). Chemosensory neurons with overlapping functions direct chemotaxis to multiple chemicals in C. elegans. Neuron.

[11] Bargmann CI. Chemosensation in C. elegans. WormBook. The C. elegans Research Community. 2006. doi:10.1895/wormbook.1.7.1. 10.1895/wormbook.1.123.1 PMC4781564 18050433

[12] Pierce-Shimomura JT, Morse TM, Lockery SR (1999). The fundamental role of pirouettes in Caenorhabditis elegans chemotaxis. J Neurosci.

[13] de Bono M, Maricq AV (2005). Neuronal substrates of complex behaviors in C. elegans. Annu Rev Neurosci.

[14] Sengupta P, Samuel AD (2009). Caenorhabditis elegans: a model system for systems neuroscience. Curr Opin Neurobiol.

[15] Carvalhal Marques F, Volovik Y, Cohen E (2015). The roles of cellular and organismal aging in the development of late-onset maladies. Annu Rev Pathol.

[16] Cronin CJ, Feng Z, Schafer WR (2006). Automated imaging of C. elegans behavior. Methods Mol Biol.

[17] Husson SJ, Costa WS, Schmitt C, Gottschalk A. Keeping track of worm trackers. WormBook. 2012. The C. elegans Research Community. doi:10.1895/wormbook.1.7.1. 10.1895/wormbook.1.156.1 PMC4781246 23436808

[18] Baek JH, Cosman P, Feng Z, Silver J, Schafer WR (2002). Using machine vision to analyze and classify Caenorhabditis elegans behavioral phenotypes quantitatively. J Neurosci Methods.

[19] Yemini E, Jucikas T, Grundy LJ, Brown AE, Schafer WR (2013). A database of Caenorhabditis elegans behavioral phenotypes. Nat Methods.

[20] Ramot D, Johnson BE, Berry TL, Carnell L, Goodman MB (2008). The Parallel Worm Tracker: a platform for measuring average speed and drug-induced paralysis in nematodes. PLoS One.

[21] Swierczek NA, Giles AC, Rankin CH, Kerr RA (2011). High-throughput behavioral analysis in C. elegans. Nat Methods.

[22] Yemini E, Kerr RA, Schafer WR (2011). Tracking movement behavior of multiple worms on food. Cold Spring Harb Protoc.

[23] Ma DK, Vozdek R, Bhatla N, Horvitz HR (2012). CYSL-1 interacts with the O2-sensing hydroxylase EGL-9 to promote H2S-modulated hypoxia-induced behavioral plasticity in C. elegans. Neuron.

[24] Salmond D (2001). Target tracking: introduction and Kalman tracking filters. IEEE International Seminar Target Tracking: Algorithms and Applications.

[25] Gomez-Marin A, Stephens GJ, Louis M (2011). Active sampling and decision making in Drosophila chemotaxis. Nat Commun.

[26] Pierce-Shimomura JT, Dores M, Lockery SR (2005). Analysis of the effects of turning bias on chemotaxis in C. elegans. J Exp Biol.

[27] Iino Y, Yoshida K (2009). Parallel use of two behavioral mechanisms for chemotaxis in Caenorhabditis elegans. J Neurosci.

[28] Stein BE (2012). The New Handbook of Multisensory Processes.

[29] Fetsch CR, DeAngelis GC, Angelaki DE (2013). Bridging the gap between theories of sensory cue integration and the physiology of multisensory neurons. Nat Rev Neurosci.

[30] Duistermars BJ, Frye MA (2010). Multisensory integration for odor tracking by flying Drosophila: Behavior, circuits and speculation. Commun Integr Biol.

[31] Dubnikov T, Cohen E (2015). Proteostasis collapse, inter-tissue communication, and the regulation of aging at the organismal level. Front Genet.

[32] Herndon LA, Schmeissner PJ, Dudaronek JM, Brown PA, Listner KM, Sakano Y, Paupard MC, Hall DH, Driscoll M (2002). Stochastic and genetic factors influence tissue-specific decline in ageing C. elegans. Nature.

[33] Stroustrup N, Anthony WE, Nash ZM, Gowda V, Gomez A, Lopez-Moyado IF, Apfeld J, Fontana W (2016). The temporal scaling of Caenorhabditis elegans ageing. Nature.

[34] Kenyon CJ (2010). The genetics of ageing. Nature.

[35] Cohen E, Bieschke J, Perciavalle RM, Kelly JW, Dillin A (2006). Opposing activities protect against age-onset proteotoxicity. Science.

[36] Lithgow GJ, White TM, Melov S, Johnson TE (1995). Thermotolerance and extended life-span conferred by single-gene mutations and induced by thermal stress. Proc Natl Acad Sci U S A.

[37] Morley JF, Brignull HR, Weyers JJ, Morimoto RI (2002). The threshold for polyglutamine-expansion protein aggregation and cellular toxicity is dynamic and influenced by aging in Caenorhabditis elegans. Proc Natl Acad Sci U S A.

[38] Sznitman R, Gupta M, Hager GD, Arratia PE, Sznitman J (2010). Multi-environment model estimation for motility analysis of Caenorhabditis elegans. PLoS One.

[39] Greenblum A, Sznitman R, Fua P, Arratia PE, Sznitman J (2014). Caenorhabditis elegans segmentation using texture-based models for motility phenotyping. IEEE Trans Biomed Eng.

[40] Branson K, Robie AA, Bender J, Perona P, Dickinson MH (2009). High-throughput ethomics in large groups of Drosophila. Nat Methods.

[41] Kabra M, Robie AA, Rivera-Alba M, Branson S, Branson K (2013). JAABA: interactive machine learning for automatic annotation of animal behavior. Nat Methods.

[42] Bargmann CI, Hartwieg E, Horvitz HR (1993). Odorant-selective genes and neurons mediate olfaction in C. elegans. Cell.

[43] Chalasani SH, Chronis N, Tsunozaki M, Gray JM, Ramot D, Goodman MB, Bargmann CI (2007). Dissecting a circuit for olfactory behaviour in Caenorhabditis elegans. Nature.

[44] Sengupta P, Chou JH, Bargmann CI (1996). odr-10 encodes a seven transmembrane domain olfactory receptor required for responses to the odorant diacetyl. Cell.

[45] Zaslaver A, Liani I, Shtangel O, Ginzburg S, Yee L, Sternberg PW (2015). Hierarchical sparse coding in the sensory system of Caenorhabditis elegans. Proc Natl Acad Sci U S A.

[46] Gray JM, Hill JJ, Bargmann CI (2005). A circuit for navigation in Caenorhabditis elegans. Proc Natl Acad Sci U S A.

[47] Piggott BJ, Liu J, Feng Z, Wescott SA, Xu XZ (2011). The neural circuits and synaptic mechanisms underlying motor initiation in C. elegans. Cell.

[48] Kocabas A, Shen CH, Guo ZV, Ramanathan S (2012). Controlling interneuron activity in Caenorhabditis elegans to evoke chemotactic behaviour. Nature.

[49] Haralick R, Shapiro L (1992). Computer and Robot Vision.

[50] Horn B (1986). Robot Vision.

[51] Anderson TW (1958). Introduction to Multivariate Statistical Analysis.

