# Supplementary material for: A multi-animal tracker for studying complex behaviors
Source: BMC Biol. 2017 Apr 6;15:29. doi: 10.1186/s12915-017-0363-9 (PMC5383998; doi:10.1186/s12915-017-0363-9)
Supplement: Supplementary file 8 — The Zaslaver’s lab Multi-Animal Tracker (MAT) - a user manual. (PDF 863 kb) [file 12915_2017_363_MOESM1_ESM.pdf]

The Zaslaver's Lab Multi-Animal Tracker (MAT) V2.0 User manual

February 8, 2017

Contents

1 About this Manual **2**

2 General **2**
2.1 The Multi-Animal Tracker Suite 2
2.2 The AnimalsRecorder Module 2
2.3 The AnimalsTracker Module 2
2.4 The Analyses Plugins 2

3 Prerequisites **3**
3.1 Software and Hardware Requirements 3
3.2 Video prerequisites 3

4 Installation **3**

5 Examples **4**
5.1 Training the Tracker and Tracking Worms 4
5.2 Exploration and Tracks Analysis 7
5.2.1 Saving the Tracks for Further Analyses 7
5.2.2 Displaying tracks 8
5.2.3 Saving tracked videos 9
5.3 Speed Analysis 9
5.4 Using the Analyses Plugins module 11
5.4.1 Polygon Dynamics 11
5.4.2 Attraction Vector Fields 12

6 Miscellaneous **13**
6.1 Batch work 13
6.1.1 Batch tracking in parallel 14

7 License Information **14**

1 About this Manual

This manual intends to provide new users with a detailed description on how to use the Zaslaver Lab's Multi-Animal Tracker. It covers both the installation process and some basic usage examples. It is recommended to follow the *examples* section successively as some examples are based on the previous ones.

2 General

The Multi-Animal Tracker is a *MATLAB*® (© The MathWorks, Inc., http://www.mathworks.com) code library that provides a suite of tools for studying the behavior of small model organisms. Note: the examples provided here refer to tracking worms but any animal model can be tracked using this system.

2.1 The Multi-Animal Tracker Suite

The Multi-Animal Tracker suite includes:

- AnimalsRecorder - A matlab interface for video acquisition.
- AnimalsTracker - Uses recorded video to track animal movement, and produces *tracks* objects that can later be analysed.
- Analyses Plugins - Plugins for out-of-the-box analyses of animal motility.

2.2 The AnimalsRecorder Module

The AnimalsRecorder module provides an interface for the acquisition of behavioral assay videos. It uses the uncompressed Motion Jpeg 2000 (mj2) to store the acquired videos. Parameters such as frame rate and exposure can be easily controlled.

2.3 The AnimalsTracker Module

The AnimalsTracker uses the behavioral video as an input file and extracts the tracks of moving animals. To correctly identify animals, and avoid the tracking of irrelevant objects, the tracker should be *trained*.

2.4 The Analyses Plugins

We provide a small library of out-of-the-box analysis tools for extracted trajectories. For instance, the polygonDynamics function can be used to mark an area in the acquisition field and plot the number of animals that enter and leave that field of interest over time. We encourage the users of this tracker to share their code for trajectories analyses with others.

3 Prerequisites

3.1 Software and Hardware Requirements

- MATLAB (tested on 2014a, 2014b, 2012b). The following package should also be installed:
  - MATLAB Image Processing package.
- At least 8GB of memory.
- Tested on Windows 7, 8, 10, and various Linux distributions.

For the AnimalsRecorder module:

- A Mathworks supported camera[1].
- MATLAB Image Acquisition package.

3.2 Video prerequisites

The tracker can be used to extract trajectories from most common video types (there is no requirement to acquire movies with the AnimalsRecorder module). For accurate tracking, it is best to have:

- A frame rate of 1Hz or higher.
- A synchronized population of animals (or a population with small variance in shape and size).

The tracker has been tested with worms occupying a minimum area of ~ 30 pixels.

4 Installation

- Extract the contents of the archive file *MultiAnimalTrackerSuite.zip* into a new directory of choice on your computer.
- Open MATLAB and navigate to the new directory.
- Right click on the directory. Go to the *Add to Path* menu, and click on *Selected Folder and Subfolders.*

[1]Check Mathworks supported vendors online.

Figure 1: Adding the MAT root directory to the path.

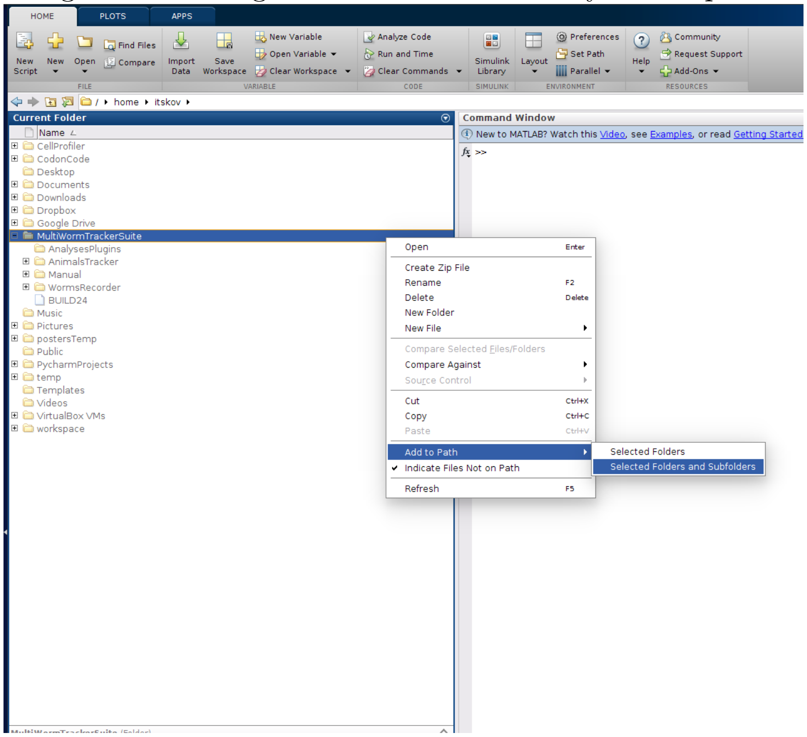

5 Examples

In the installation folder, under the *SampleVideo* directory, we provide a sample video (*SampleMovie.avi*) which we will use for the following examples.

5.1 Training the Tracker and Tracking Worms

- Make sure you performed the installation steps mentioned in the *Installation* section.
- In the MATLAB Command Window, type: *AnimalsTracker*

```
fx >> AnimalsTracker
```

- A graphical user interface will appear. Go to the *file* menu, click *open*, and choose the *SampleMovie.avi* that appears in the *SampleVideo* directory of the Multi-Animal Tracker root installation folder.

Figure 2: The tracker's graphical user interface

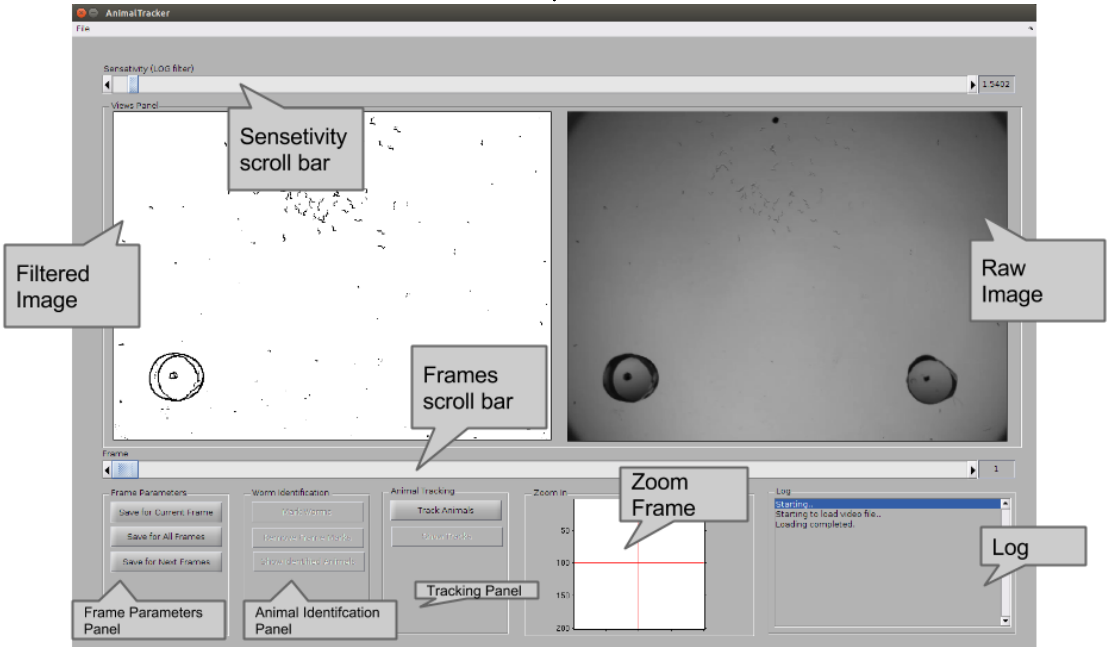

- The filtered frame may show the worms' shapes as round blobs. To change this, drag the *sensitivity scroll bar* leftwards to increase filtering sensitivity or rightwards to decrease it until you can easily see the correct outlines of the worms, without getting a lot of entities which are not worms (this will occur if the sensitivity is too high). For the provided sample the optimal sensitivity is around 1.5.

- To save the chosen sensitivity threshold and to apply it to the entire movie, press on the *Save For All Frames* button in the *Frame Parameters* panel.

- Now that the sensitivity is set for all frames we can start training the tracker to identify the animals (worms in this case):
  - Press the *Mark Worms* button in the *Worm Identification panel*. The mouse cursor will change into a crosshair.
  - Click on worms[2] in the *Filtered Image* frame. You may use the *Zoom Frame* when the worms appear too small in the *Filtered Image* frame. Correctly marked worms will appear green.
  - When you wish to stop marking worms, **right** click somewhere in the window. The log window keeps tracks of marked worms.

[2]Avoid marking entities which are not single animals (i.e animal collisions or aggregates).

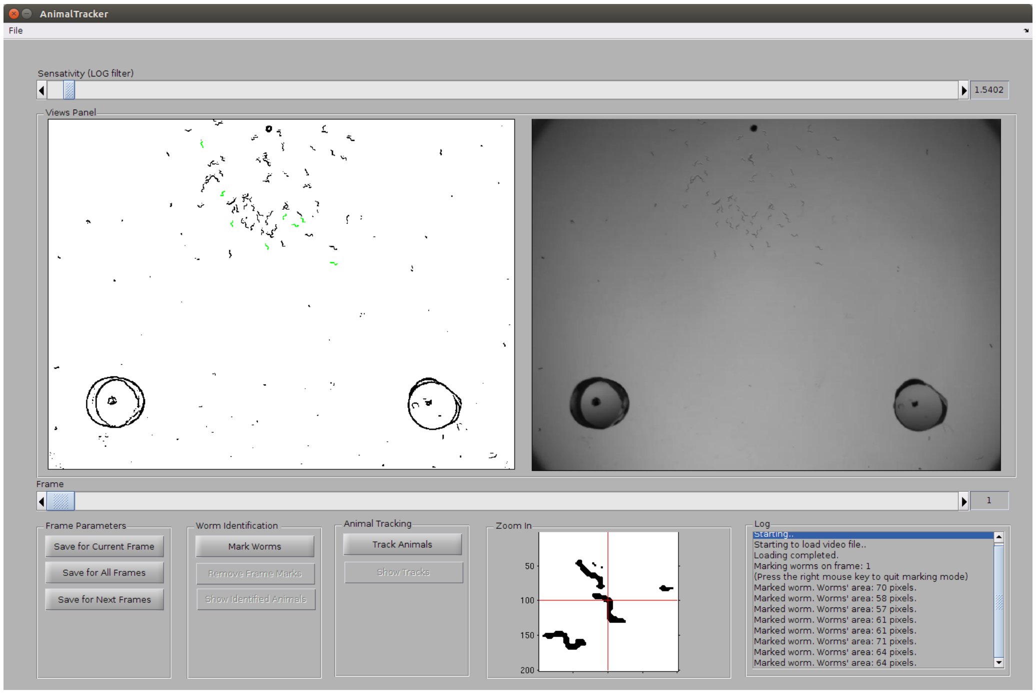

- Now, use the *Frame scroll bar* to move to a different point in the video, and repeat the previous step (press *Mark Worms* and click on a few worms). Go on and repeat this step for a couple of time points throughout the video.

- To verify that the tracker is well-trained, choose a frame randomly. Then press on the *Show Identified Animals* button (Worm Identification Panel). The entities that were identified as worms should be marked blue (or cyan, if they were also marked by you). If the tracker failed to mark some worms correctly, press the *Mark Worms* button again, and mark the worms that it failed to identify.

- When satisfied with the accuracy of the identification, proceed with the tracking[3] by clicking on the *Track Animals* button in the *Animal Tracking* panel. This process may take a few minutes, during which messages will be printed in the main *MATLAB* window. Once finished, a message will be printed in the *log* frame.

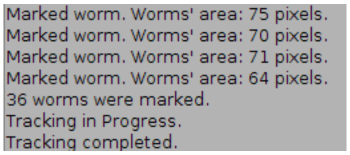

- Once the tracking is complete, you may press the *Show Tracks* button in the *Animal Tracking* panel to see the tracking results.

[3]to perform batch tracking, see the *Miscellaneous* section.

Figure 3: Viewing the tracks after the tracking procedure is completed.

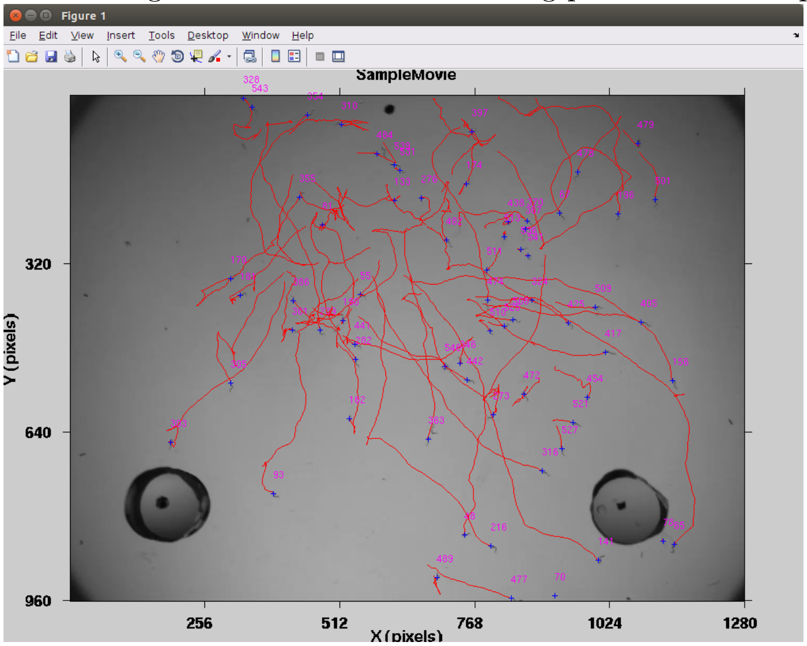

5.2 Exploration and Tracks Analysis

5.2.1 Saving the Tracks for Further Analyses

To save the tracks extracted by the tracker, go to the *File* menu and click on *Save Tracks*. You will be asked to choose a location in which a **.mat* file will be saved. This file contains all of the information extracted from the video. Next, go back to the *MATLAB* main window, and find the file you have just saved on the directory tree. **Double clicking** on that file will introduce two new variables into your *MATLAB* workspace: *tracker* and *tracks*.

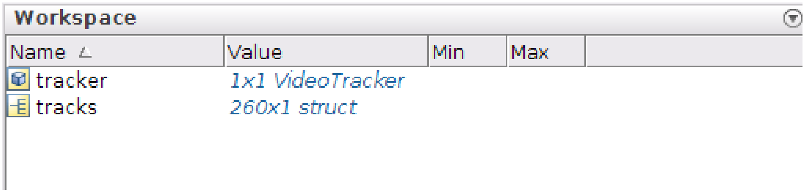

The *tracks* variable is an array of all tracks acquired from the movie. The most important feature of each track is its *path*, or the array of x,y values for the entity at each frame it was recorded. (See *Speed Analysis*.) The *tracker* is the object that holds the information and functionality required for the exploration and analysis of the tracks. For instance,

```
>> tracker.numberOfFrames
```

will output the number of frames in the tracked video, and

```
>> tracker.tracks(54).numberOfSteps
```

will output the number of steps in track number 54 in the tracks list.

5.2.2 Displaying tracks

The *tracker* object allows displaying the trajectories of the extracted tracks. For example, the following command,

```
>> tracker.viewTracks(tracks,[1, tracker.numberOfFrames]);
```

will display all tracks between frame 1 and the end of the video.

Each track is given an ID. The ID is printed next to the worm when viewing the tracks using the *tracker.viewTracks* command[4].

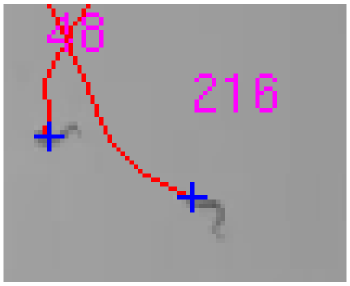

You can use the *viewTracks* command to view specific tracks based on their tracks ID. For example,

```
>> tracker.viewTracks(tracks([tracks.id] == 216), [1, tracker.numberOfFrames])
```

will display only the track which ID is 216.

[4]Keep in mind that its position in the list is not necessarily its ID.

Figure 4: Choosing tracks based on their track ID

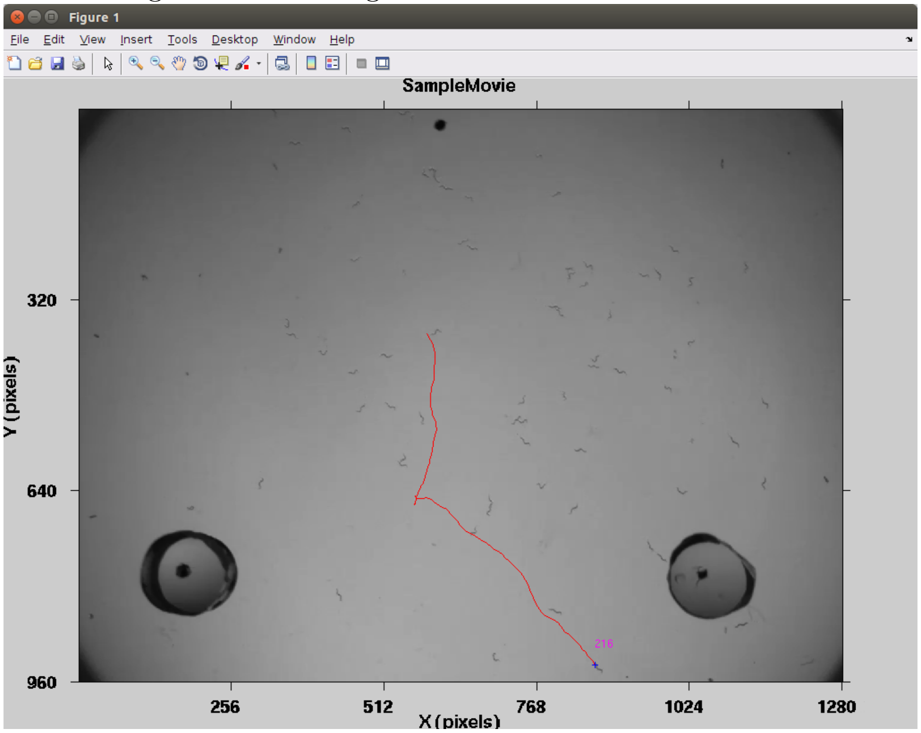

5.2.3 Saving tracked videos

It is possible to save the videos with the tracked trajectories on top of it. This can easily be done with the same *viewTracks* method. The following command,

```
>> tracker.viewTracks(tracks,[1, tracker.numberOfFrames],0,'c:\MyVideos\')
```

will save the tracked video into *MyVideos* directory[5]. The third parameter passed to the *viewTracks* method tells the tracker whether or not to show the worms' center of mass. Setting it to 1 will show the center of mass as a green cross.

5.3 Speed Analysis

Each *track* in the *tracks* array contains multiple fields. The *path* field holds a list of coordinates that describe the track's trajectory over time. The track's *path* field contains 4 columns:

1. A serial frame number for this particular point.
2. The X coordinate.
3. The Y coordinate.

[5]To batch track videos, see *Miscellaneous*

4. A boolean value that indicates whether this point was observed or predicted (the tracker tries to predict track positions once it loses them).

The following code,

```
>> track216 = tracks([tracks.id] == 216)
>> plot(track216.path(:,2), track216.path(:,3),'r')
```

will plot[6],

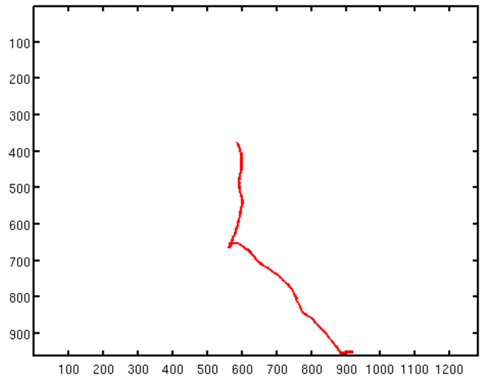

We can calculate the size of each step in each time point, and average it over the entire video to come up with the mean speed of the worms in the current assay.

```
>> allSpeeds = [];
>> for t=tracker.tracks
>> trackSteps = diff(t.path(:,2:3));
>> trackSpeeds = sqrt(trackSteps(:,1).^2 + trackSteps(:,2).^2);
>> allSpeeds = [allSpeeds; trackSpeeds];
>> end
```

Before we continue, it would be a good idea to get rid of outliers that might be due unresolved collisions.

```
>> allSpeeds = allSpeeds(allSpeeds < quantile(allSpeeds,0.99))
```

And now we can plot, for instance, the velocity histogram,

```
>> hist(allSpeeds);
```

[6]Since the origin of the axis in Matlab images is different from a Matlab plot, the following code will actually plot a mirror image. To solve this, execute *set(gca,'YDir','reverse')* prior to the *plot* command.

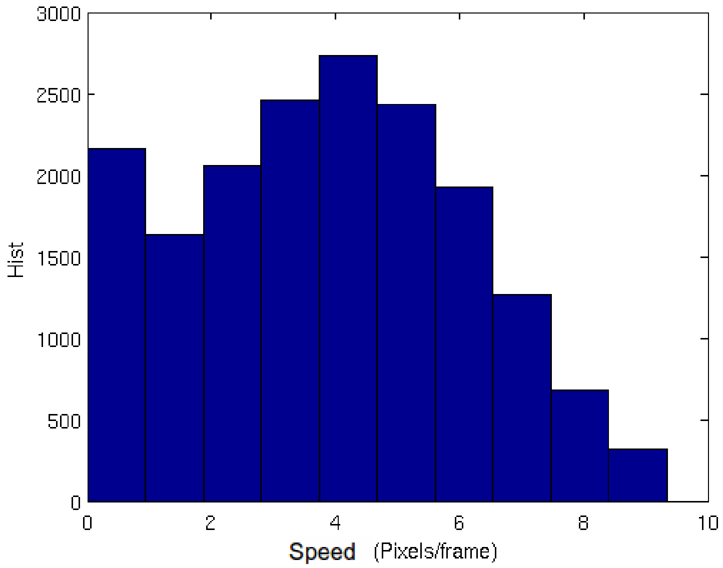

In this assay, the speed grossly distributes around 4 pixels/frame. However, a close-to-zero speed is also quite common.[7]

5.4 Using the Analyses Plugins module

We provide the following analyses tools as part of the *Analyses Plugins* library.

- PolygonDynamics - Allows marking a polygon within the video field, and plots the number of animals that enter and leave the polygon as a function of time.
- AttractionVectorField - Plots a vector field to visualize animal direction and density.

5.4.1 Polygon Dynamics

Execute the following command in MATLAB command window:

```
>> polygonDynamics(tracker,1);
```

The first frame of the video should appear, and you'll be asked to mark a polygon of interest on the image. Use the left mouse key to mark points, and the right mouse key to stop the marking. Mark, for example, around the chemoattractant point (right circle-shaped entity on the *SampleVideo*).

[7] It is a good idea to first eyeball the tracks to make sure that zero speeds are not due to static pieces of dust or other debris that were erroneously identified as animals.

Figure 5: Marking a polygon of interest.

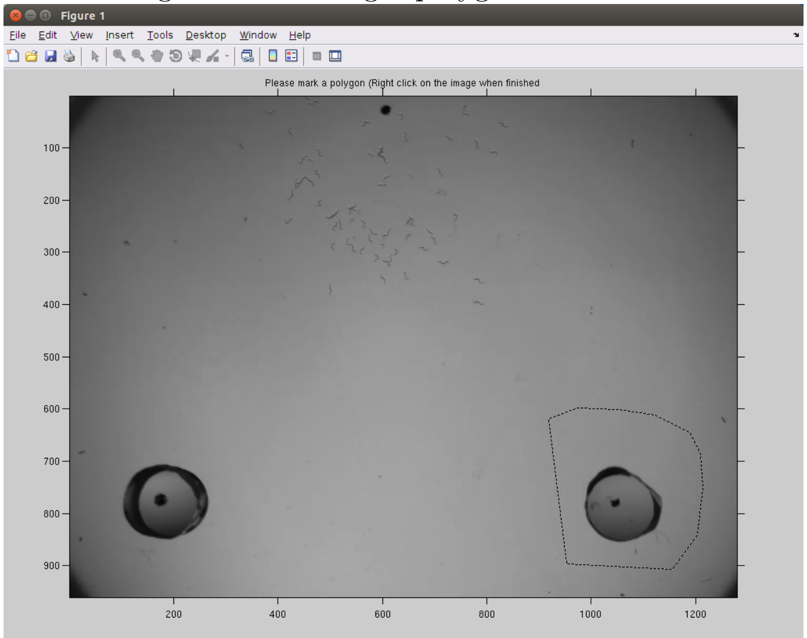

Once you're done marking the polygon, a curve will appear showing the number of worms that have entered/left the polygon as a function of time:

Figure 6: Flux of worms through the polygon of interest.

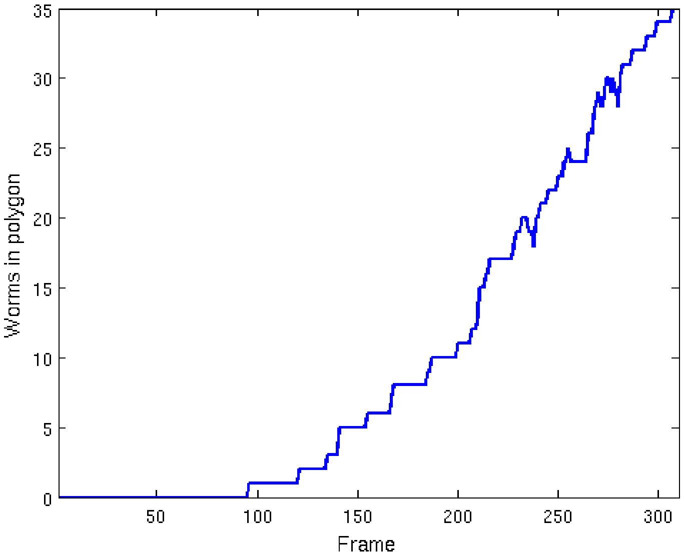

5.4.2 Attraction Vector Fields

The following command,

```
>> attractionVectorField(tracker)
```

will plot a vector field that shows the average direction and number of worms in different areas of the field. For the provided *SampleVideo* the result would be,

Figure 7: Worm directionality vector field.

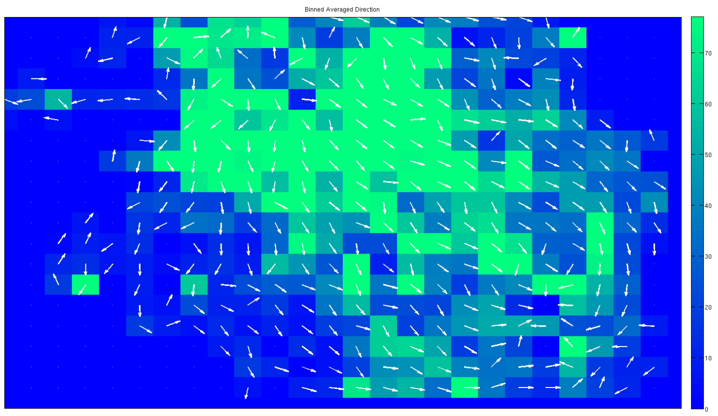

6 Miscellaneous

6.1 Batch work

It may be convenient to process multiple experiments at once. Included in the *AnalysesPlugins* folder are three files to batch track animals and create videos: FileLister, batchCreateMovies, and trackerCrawler. Install these as you would the rest of the Analysis suite.

- To batch track animals, open the AnimalsTracker and train the tracker as described in *Examples*. When you are done, however, instead of clicking on *Track Animals*, go to the "File" menu and click on "Save Statistics". Save this file in the same folder as the video being tracked.
- Repeat the previous step for all of your behavioral videos.
- To batch track these videos, type the following into Matlab:

  ```
  >> trackerCrawler( sourceDirectory, targetDirectory )
  ```

  Where *sourceDirectory* is the directory/folder of the saved statistics and videos and *targetDirectory* is the location you wish to save the tracks in. When the tracking begins for each video, a new folder will be created to hold the *tracks* file.

- Once you have the tracks[8] you can also batch create movies by running the command

  ```
  >> batchCreateMovies( sourceDirectory )
  ```

6.1.1 Batch tracking in parallel

If you have a CPU with multiple cores, it is also possible to track multiple files at once. To do so, in the main matlab window, type in

```
>> matlabpool #
```

where # is the number of cores you wish to devote to this task. You can then run the *trackerCrawler* command as usual.

7 License Information

This library is provided as is under the GNU General Public License v3.

[8]You can save time by sequentially tracking and then creating videos by separating their commands with a semicolon. For instance, if the "current folder" in matlab is the location of the video files and statistics, you can simply run the command "trackerCrawler('.','.'); batchCreateMovies('.');" which will create videos only after the tracking is done.
