# Supplementary figures and images for: A multi-animal tracker for studying complex behaviors
Source: BMC Biol. 2017 Apr 6;15:29. doi: 10.1186/s12915-017-0363-9 (PMC5383998; doi:10.1186/s12915-017-0363-9)

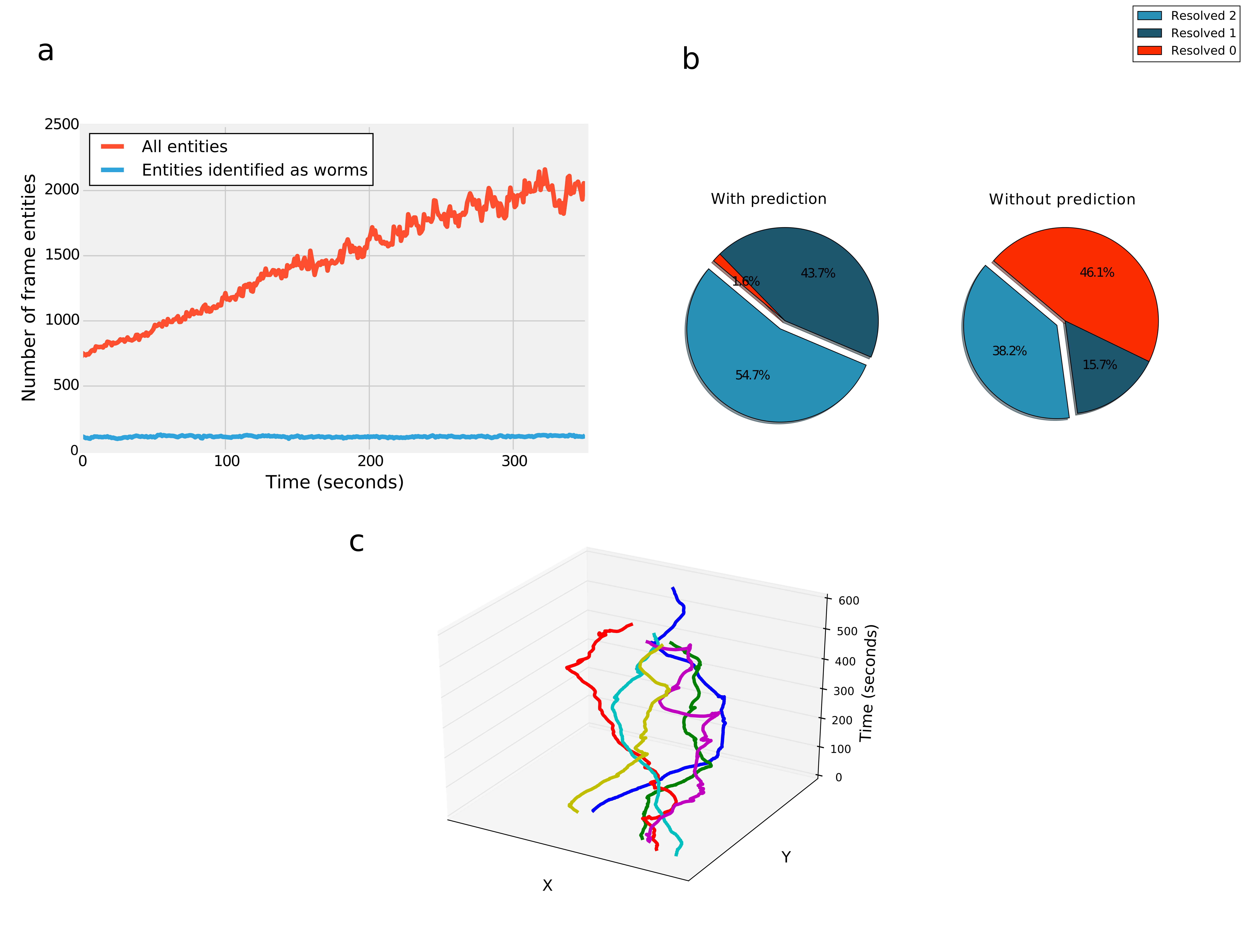

Supplement: Supplementary file 9 — The tracker implements a machine learning algorithm and a predictor that contribute to accurate detection and precise tracking following collision. (a) In a typical long behavioral assay, condensation on the lid and trail marks left behind significantly contribute to entities discovered by the tracker. The number of these entities increases with time (red curve), but since the tracker employs a machine learning algorithm for animal identification, it ignores these erroneously detected entities, and the number of genuine animal entities remains constant throughout the experiment (blue curve). (b) The software implements a predictor that aids resolving animals following collisions. The colors/numbers correspond to the number of animals resolved following collision of two animals: 2 – both animals resolved; 1 – only one animal resolved; 0 – none. (c) An example of six fully-extracted tracks of six individual worms during 10 minutes of tracking. Overall, approximately 100 worms were loaded on the plate, and the tracker software can often keep track of individual animals despite frequent collisions. (TIF 1660 kb) [file 12915_2017_363_MOESM6_ESM.tif]

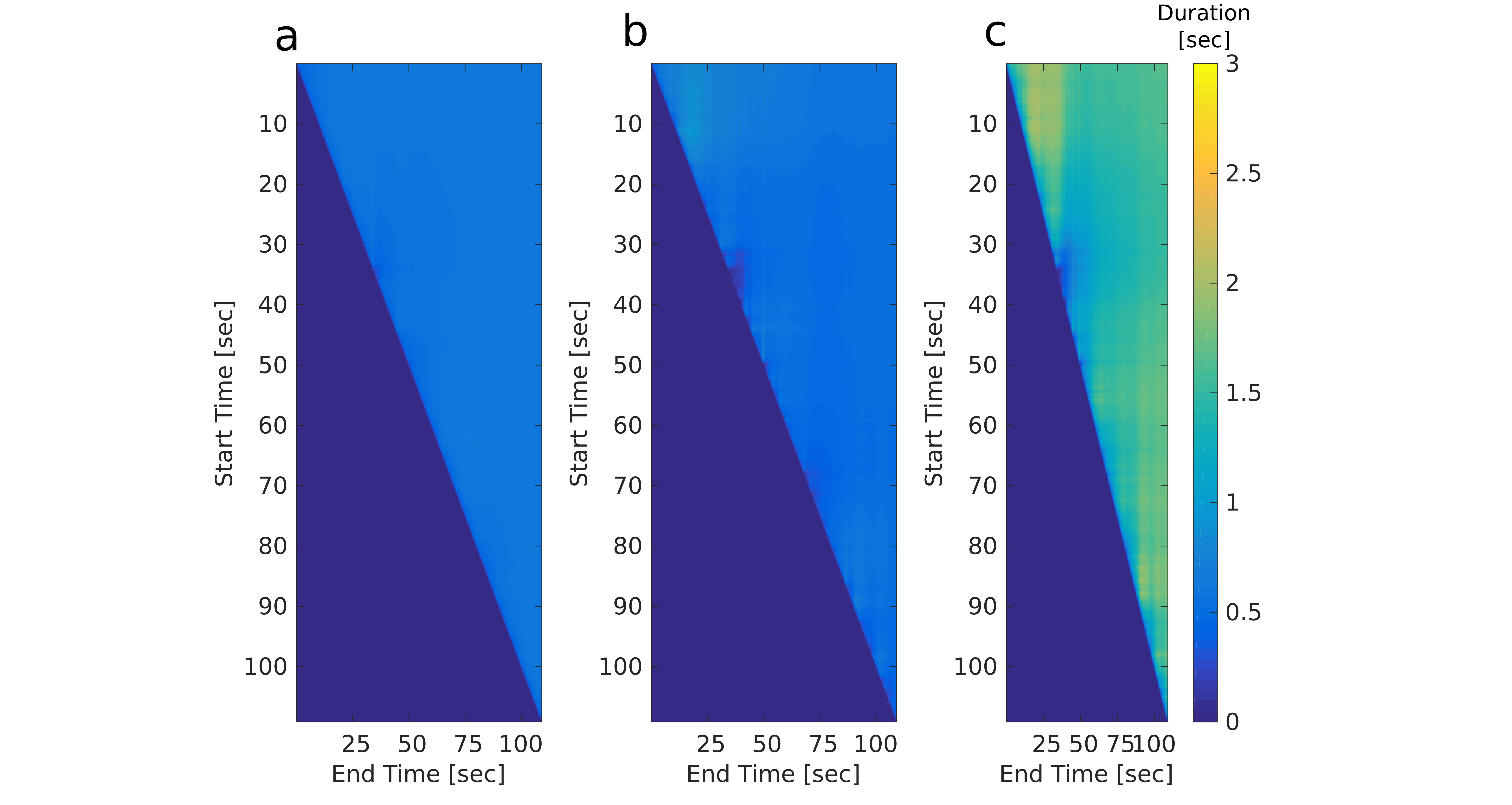

Supplement: Supplementary file 10 — Employing both the machine learning (ML) algorithm and the Kalman-type predictor significantly improves accurate animal detection and tracking. This is true even when analyzing a fairly easy movie with clear contrast between animals and background. We analyzed a crowded arena of swimming zebrafish in three different ways: (a) disabling both ML segmentation and the predictor; (b) applying ML segmentation but disabling the predictor; and (c) applying both ML segmentation and the predictor in the segmentation and tracking algorithm. To provide a quantitative measure for the advantage using both the ML and the predictor we calculated tracks’ length (in seconds) as extracted throughout the movie. Each point (i,j) in the heat map represents the average track length between time i (y-axis) and time j (x-axis). We then identified the segments with the longest tracks in each of these three-way analyses, and averaged them: (a) 0.6 ± 0.02 [s]; (b) 1.15 ± 0.14 [s]; (c) 2.2 ± 0.3 [s]. Thus, employing both the ML and the predictor improves track length by more than three-fold. The movies corresponding to these three-way analyses are provided as Additional file 8: Movie S5, Additional file 9: Movie S6, and Additional file 10: Movie S7, corresponding to (a), (b), and (c), respectively. Note, in Additional file 8: Movie S5 (neither ML nor predictor), the many background entities that are being tracked and the tiny insect that was crawling on the left, which would have been part of the statistics unless ML was used. (TIF 979 kb) [file 12915_2017_363_MOESM7_ESM.tif]

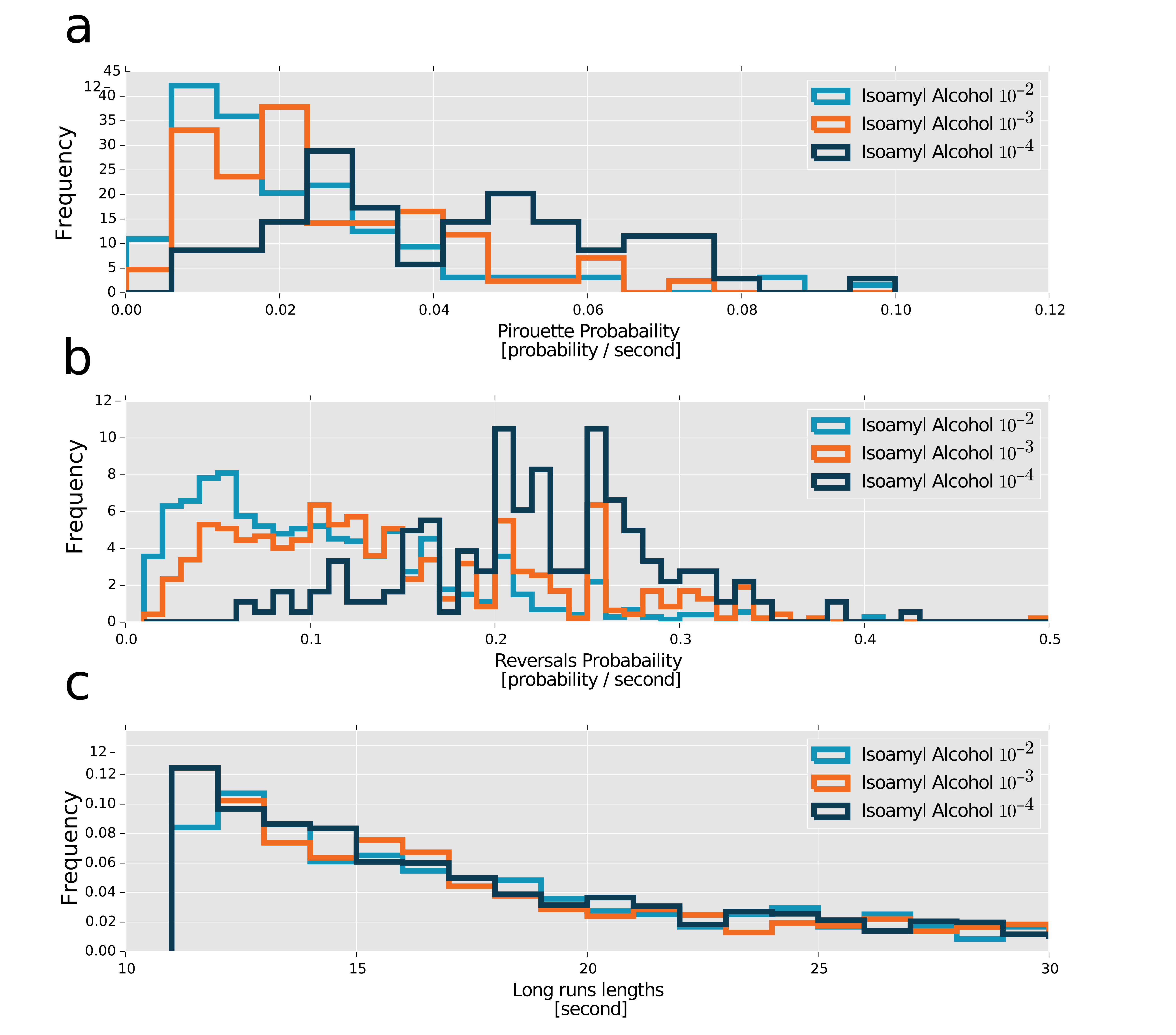

Supplement: Supplementary file 11 — Various chemotaxis parameters are dose dependent. Histograms of (a) probability for a pirouette, (b) probability for reversal/sharp-turn, (c) run lengths (time between reversals). (TIF 670 kb) [file 12915_2017_363_MOESM11_ESM.tif]

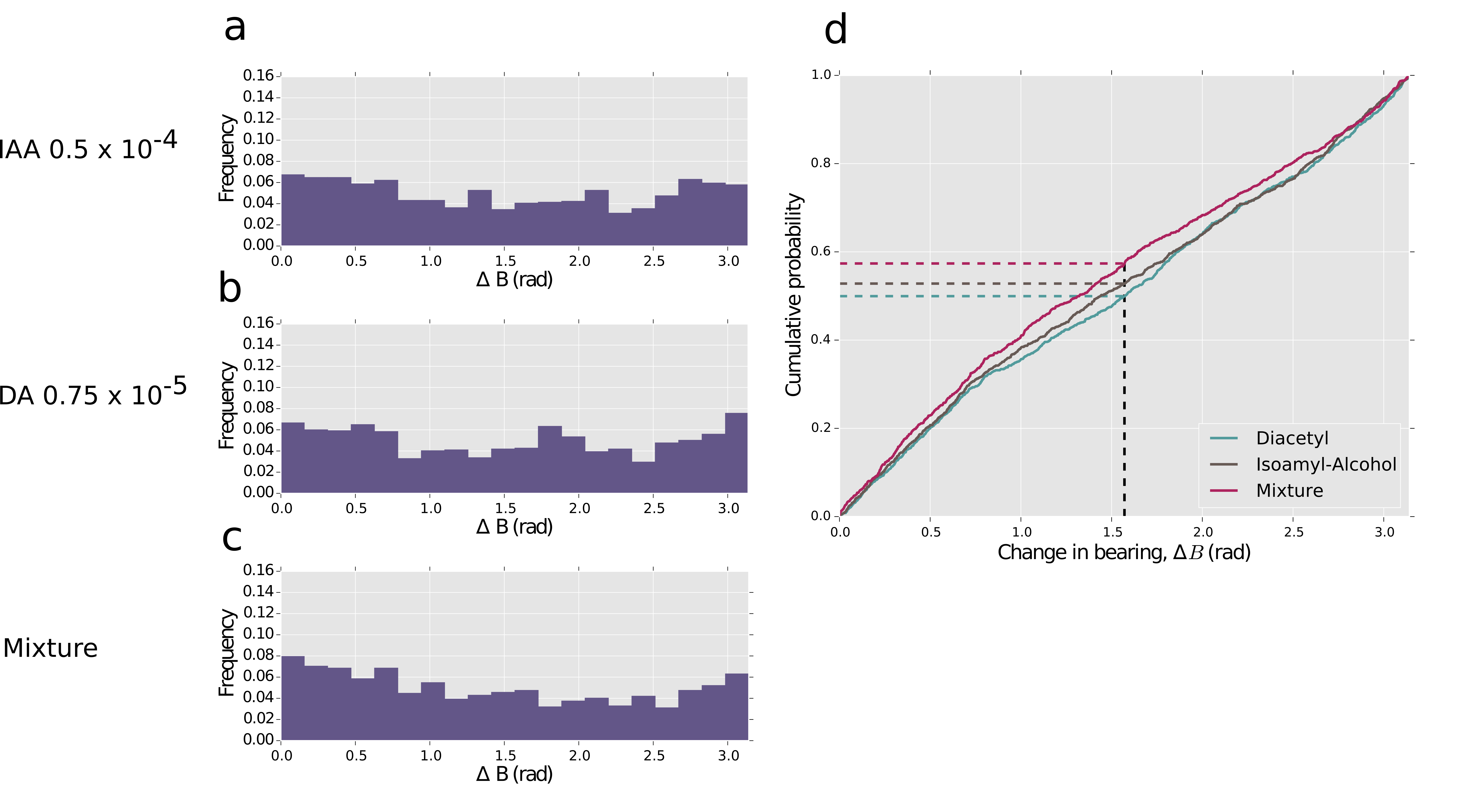

Supplement: Supplementary file 12 — Worms integrate environmental cues and enhance chemotaxis towards richer environments. When presented with a mixture of cues (c, d), worms are more likely to maintain their general direction following a pirouette (ΔB < Π/2), as compared to worms presented with each of the components separately (a, b, d; P < 0.005). We could not detect a significant increase in the probability for a larger directional change for worms that were presented with a mixture, compared to worms that were presented with single components of the mixture (P > 0.2). (TIF 943 kb) [file 12915_2017_363_MOESM12_ESM.tif]
